# Unraveling Bifurcating Pathways for CO and HCOOH Formation: Insights from Stopped-Flow FTIR Spectroscopy of a Second-Sphere Modified Mn Catalyst

**DOI:** 10.1021/jacs.5c04274

**Published:** 2025-06-18

**Authors:** Samir Chattopadhyay, Sudip Barman, Reiner Lomoth, Leif Hammarström

**Affiliations:** † Department of Chemistry—Ångström Laboratories, 8097 Uppsala University, SE 75120 Uppsala, Sweden; ‡ School of Chemical Sciences, 62397 Indian Association for the Cultivation of Science, 2A Raja SC Mullick Road, Kolkata 700032, West Bengal, India

## Abstract

Manganese bipyridine tricarbonyl complexes show high efficiency and selectivity in electrochemical CO_2_ reduction (e-CO_2_RR) to CO. Efforts to shift selectivity toward HCOOH have been made by introducing second-sphere hydroxyl or amine functional groups and using amines or proton-coupled electron transfer (PCET) mediators. However, the direct spectroscopic evidence for the bifurcation pathways leading to CO and HCOOH remained elusive. Using stopped-flow mixing with decamethyl cobaltocene reductant and time-resolved infrared (TRIR) spectroscopy, we identified, for the first time, the key intermediates in this bifurcation pathway for an Mn complex with second-sphere hydroxyl groups in real time under catalytic conditions. The measured rate constants align with reported TOF values from electrochemical studies, validating the relevance of the results to e-CO_2_RR conditions. Our findings reveal that HCOOH production involves proton transfer from hydroxyl groups to the doubly reduced Mn center, forming the Mn-hydride intermediate, followed by CO_2_ insertion, leading to the Mn-formate intermediate. However, the inability of the resulting phenolate to rebind protons from weak acids like water leads to rapid catalyst degradation, limiting sustained catalysis. This work provides mechanistic insights and paves the way for designing molecular catalysts with enhanced selectivity and stability for HCOOH production during e-CO_2_RR.

## Graphical abstract

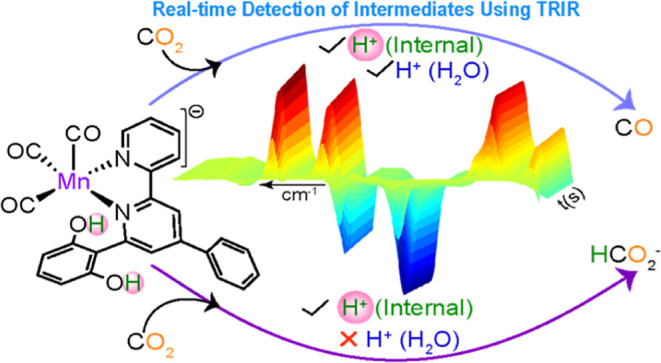

## Introduction

Electrochemical and photochemical CO_2_ reduction reactions (e-CO_2_RR and photo-CO_2_RR, respectively) offer promising approaches to mitigating CO_2_ emissions by producing valuable products like CO and HCOOH.^1–3^ Among various transition metal-based molecular e-CO_2_RR catalysts,^4,5^ *fac*-manganese (Mn) bipyridine tricarbonyl complexes have gained attention as earth-abundant analogs of Re-based catalysts, demonstrating high efficiency, low overpotentials, and selectivity for CO formation.^6^ Unlike Re catalysts, Mn-based systems rely heavily on external proton sources, and their product selectivity can shift from CO to HCOOH under photo-CO_2_RR conditions.^7–11^ Recent strategies to enhance HCOOH production during e-CO_2_RR with Mn catalysts include incorporating second-sphere amine or hydroxyl functional groups, adding amine-based additives, or proton-coupled electron transfer (PCET) mediators.^12–16^ While prior studies using electrochemical, FTIR-spectroelectrochemistry (FTIR-SEC), and density functional theory (DFT) approaches have highlighted the role of these modifications, direct spectroscopic evidence of intermediates beyond the first species (**2** ^**–**^, Figure 1) has been rare due to challenges such as simultaneous intermediate accumulation, time resolution of FTIR-SEC, and the more positive reduction potentials of certain intermediates that often cause their rapid further reduction at the electrode, making their detection difficult.^17,18^ Therefore, most intermediates in e-CO_2_RR studies have been detected under non-catalytic conditions, such as the metal-hydride intermediate (**2H**, Figure 1) observed under an argon atmosphere, which is often used to infer the formate formation via the metal-formate intermediate (**6**, Figure 1).^15,19,20^ Transient IR spectroscopy (TRIR) combined with stopped-flow mixing has proven effective in overcoming these limitations and has been used to investigate the competitive CO_2_ and H^+^ binding kinetics to **2**_**Re**_^**–**^.^21,22^ Recently, this technique revealed a transient intermediate, **2CO**_Re_, with a half-life of 55 ms, which is proposed to play a critical role in the product release step of both e-CO_2_RR and photo-CO_2_RR catalyzed by Re catalysts.^23^

**Figure 1:**
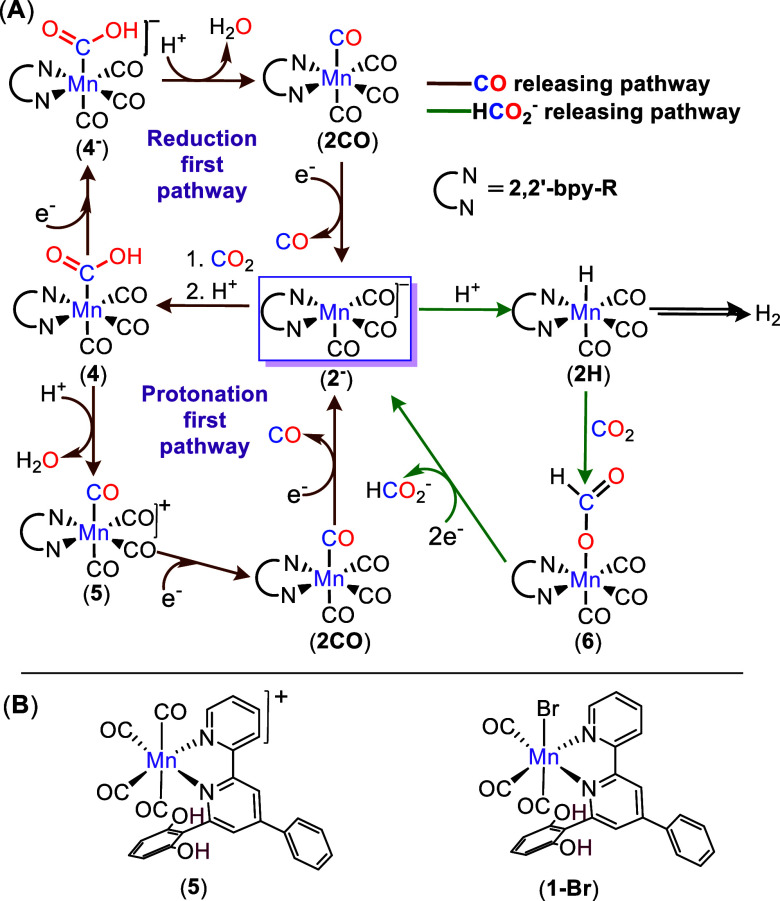
(A) The proposed mechanistic cycle of e-CO_2_RR catalyzed by Mn­(bpy-R)­(CO)_3_X complex; the intermediate at the bifurcation point of the CO and formate pathways, [Mn­(bpy-R)­(CO)_3_]^−^, **2** ^**–**^, is highlighted in a purple box. (B) Chemical structures of the complexes discussed in this paper.

The Mn catalyst, **1-Br** (Figure 1), featuring the second-sphere resorcinol group, was the first of its kind to achieve a reasonable faradaic efficiency (FY) for HCOOH production (22% HCOOH, 70% CO) during e-CO_2_RR in anhydrous acetonitrile.^12^ However, the catalyst showed rapid degradation during control potential electrolysis (CPE), the cause of which remains unclear. Upon adding 5% H_2_O, a significant shift in product selectivity was observed (FY_CO_ = 90%, FY_HCOOH_ = 4%),^24^ but the mechanism behind this change was not previously understood. To address these gaps, we aim to investigate the bifurcation pathways for CO and HCOOH formation by identifying intermediates and analyzing their kinetics under conditions relevant to e-CO_2_RR.

Herein, we present detailed mechanistic and kinetic studies of this bifurcation pathway by mixing the catalyst with reductant (CoCp_2_*) using stopped-flow and monitoring the reaction with TRIR. The similarity of our rate constants to turnover frequencies (TOFs) under e-CO_2_RR conditions suggests that the mechanisms are the same under both conditions, and that the reduction steps are not rate-limiting. Starting from the oxidized Mn-tetracarbonyl complex (**5**, Figure 1B), which exhibits similar electrochemical behavior and product selectivity to **1-Br** but at less cathodic potentials (∼100 mV), we detected several intermediates responsible for HCOOH and CO production, along with their kinetics. Binding of CO_2_ to the intermediate species **2** ^**–**^, followed by protonation, generates the carboxylic acid species **4** that leads to CO formation. In parallel, intramolecular proton transfer from an –OH group to the same intermediate **2** ^**–**^ generates the metal hydride intermediate, **2H-H^+^**, which binds CO_2_ to form the metal-formate species, **6-H^+^**, in the HCOOH formation pathway. The inability of the resulting phenolate group to reprotonate even in the presence of 5% H_2_O was identified as the key factor contributing to the catalyst degradation. Our findings also highlight the role of H_2_O in accelerating the CO and HCOOH formation pathway to a different extent, which might explain the shift in product selectivity. These insights provide a foundation for designing advanced molecular e-CO_2_RR catalysts with improved selectivity and stability toward HCOOH production.

## Results and Discussion

Complex **5** was synthesized from **1-Br** following the similar procedure reported for the [Mn­(bpy)­(CO)_4_] SbF_6_ complex.^25^ **5** was characterized by NMR, HRMS, and FTIR (Figure S1). Cyclic voltammetry showed that complex **5** exhibits similar electrochemical responses to those of **1-Br** in argon and CO_2_ atmospheres, albeit at 100 mV less cathodic potential (Figure 2). CPE experiments yielded FYs of 68% for CO and 17% for HCOOH (Figures S3–S6), indicating that replacing the axial -Br with CO does not affect product selectivity in anhydrous acetonitrile. This behavior is consistent with observations for the related Mn complex with unsubstituted bipyridine ligands.^25^

**Figure 2:**
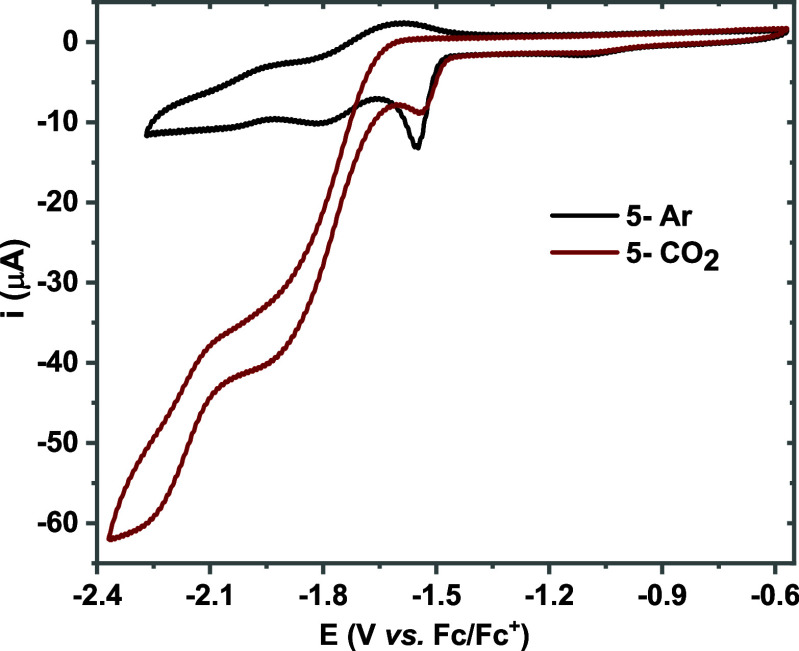
Cyclic voltammograms of complex **5** (1 mM) in acetonitrile under argon (black) and CO_2_ (brown) atmosphere. Scan rate: 100 mV/s, working electrode: glassy carbon, counter electrode: Pt. Here, tetra-butyl ammonium hexafluoro phosphate was used as a supporting electrolyte.

### TRIR Experiments Identifying **2** ^**–**^ Intermediate

Stopped-flow TRIR experiments (Schematic, Figure S6) were conducted using 0.5 mM of **5** and 2.6 mM of CoCp_2_* in a saturated CO_2_ solution (at 1 atm CO_2_) in anhydrous acetonitrile. The stated concentrations correspond to those at the FTIR observation cell. The TRIR spectra (Figure 3A) reveal two strong υ̅_CO_ bands at 1916 and 1822 cm^–1^ present already at *t* = 0 (within the time resolution of the experiment, ≈11.3 ms), consistent with the reported υ̅_CO_ values for the [Mn­(bpy)­(CO)_3_]^−^ intermediate (**2** ^**–**^, Figure 1),^9^ i.e. the intermediate at the bifurcation point of the reaction scheme in Figure 1 (marked in purple box). However, the protonation state of the hydroxyl groups remains uncertain, as they were previously observed to deprotonate under reducing potentials during FTIR-SEC experiments under an argon atmosphere.^24^ A 5–7 cm^–1^ redshift in the highest vibrational band has previously been attributed to the single deprotonation of [Re­(4-dhbpy)­(CO)_3_Cl], forming [Re­(4-dhbpy-H^+^)­(CO)_3_Cl]^−^.^26^ Complex **1-Br** exhibits similar trend upon deprotonation, with the five-coordinate intermediate **2** ^**–**^ **-H** ^**+**^ (containing a deprotonated O–H group) showing vibrational bands at 1910 and 1818 cm^–1^ during previous FTIR-SEC experiments under argon.^24^ Based on this information, the species having υ̅_CO_ at 1916 and 1822 cm^–1^ was assigned to **2** ^**–**^ with two intact O–H groups (Figure 4C), further corroborated by DFT-calculated vibrational frequencies (Table ST1). The reactivity of **2** ^**–**^ with CO_2_ and protons in the HCOOH and CO formation pathways, along with the corresponding intermediates, is discussed below.

**Figure 3:**
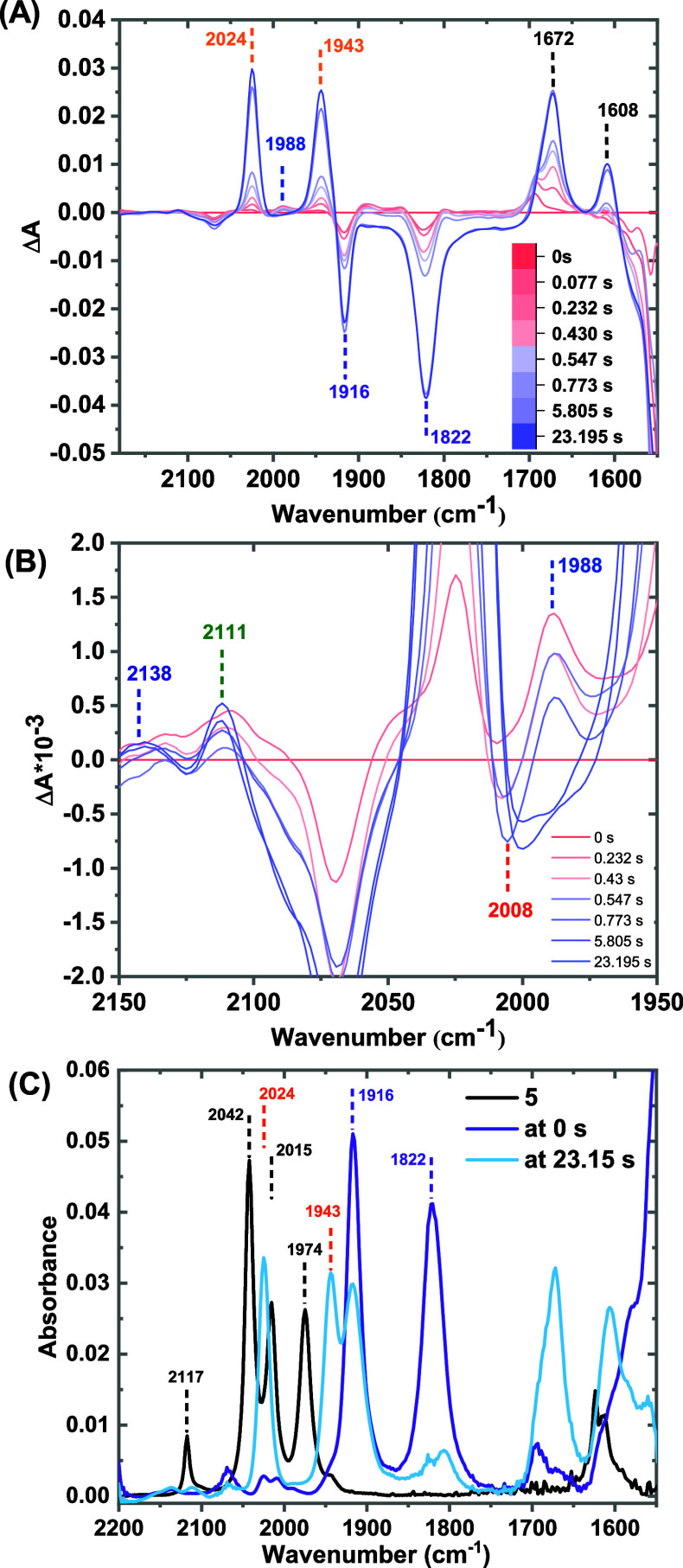
(A) TRIR difference spectra of 0.5 mM of complex **5**, showing spectral changes upon adding 2.6 mM CoCp_2_* in CO_2_-saturated anhydrous acetonitrile. (B) A zoomed-in section highlighting the appearance and disappearance of various intermediate species. In these figures, the first spectrum was taken as the background and was subtracted from the rest of the data. Therefore, the decay of the species present at the initial time (within the time resolution of the instrument) and the formation of new species are represented as negative bands and positive bands, respectively. (C) FTIR spectra showing the vibrational features of complex **5** (in black), the species present at the initial time (0 s, in red), and at 23.15 s (in sky blue) during the same stopped-flow TRIR experiment as (A). Note that the FTIR spectrum of complex **5** (in black) was obtained from a regular FTIR measurement.

**Figure 4:**
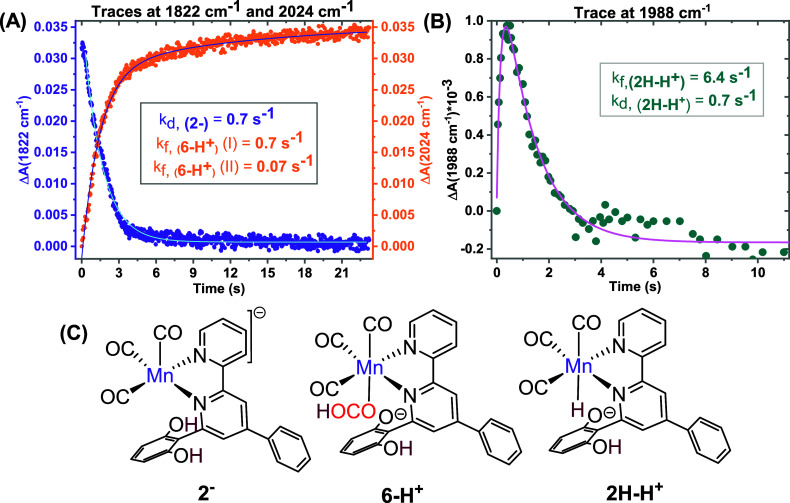
Kinetic traces of (A) **2** ^**–**^ (purple), **6-H** ^**+**^ (orange), and (B) **2H–H** ^**+**^ (turquoise) during the reaction of 0.5 mM **5** and 2.6 mM CoCp_2_* in CO_2_-saturated anhydrous acetonitrile. (B) Chemical structures of the intermediates, **2** ^**–**^, **6-H** ^**+**^, and **2H–H** ^**+**^. The rate constant values are mean values obtained from at least three individual experiments; standard deviations are given in the text.

### HCOOH Formation Pathway

The TRIR spectra reveal the accumulation and subsequent decay of a υ̅_CO_ band at 1988 cm^–1^ (Figures 3B and 4B). Previous FTIR-SEC studies under argon and DFT calculations identified this band, along with others at 1899 and 1878 cm^–1^, as signatures of the Mn-hydride intermediate with one deprotonated O–H group, **2H–H** ^**+**^ (Figure 4C).^24^ For this study, we primarily focus on the higher vibrational bands of intermediates for characterization and kinetic analysis, as the lower vibrational bands often overlap with those of other intermediates. The decay of **2** ^**–**^ (tracked at 1822 cm^–1^) was fitted by a single exponential function, yielding a rate constant of 0.70 ± 0.04 s^–1^ (Figure 4A, purple trace; uncertainties are given as ± one standard deviation). Intramolecular proton transfer from one nearby O–H group to the reduced metal center, Mn­(–I), forms the Mn-hydride intermediate (**2H–H** ^**+**^). Previous studies have shown that efficient proton transfer from the second-sphere amine or hydroxyl functional group to the reduced metal center, forming the Mn-hydride intermediate, requires a proton tunneling distance of 2.400 to 2.696 Å, characteristic of strong H-bonding.^27^ DFT geometry optimization of **2** ^**–**^ using the b3lyp functional and def2-TZVP basis set found a 2.58 Å distance between H­(OH) and the Mn­(–I) center (Figure S10), supporting the possibility of proton transfer to the reduced Mn center, forming the Mn-hydride intermediate (**2H–H** ^**+**^, Figure 1). The formation and decay kinetics of **2H–H** ^**+**^ were nicely captured using the 1988 cm^–1^ trace, showing inverted kinetics with rate constants of 6.5 ± 0.2 s^–1^ for the signal rise and 0.70 ± 0.06 s^–1^ for its decay (Figure 4B).

Figure 3 shows the appearance of a set of υ̅_CO_ bands at 2024, 1943, and 1916 cm^–1^ (Figures 3 and 4). The 1916 cm^–1^ band, less distinct in the difference spectra due to overlap with **2** ^**–**^ absorption, is visible in the absolute spectra (Figure 3C). These υ̅_CO_ values closely align with the reported values for the Mn-formate complex, [Mn­(^*t*^Bu_2_-bpy)­(CO)_3_(OCHO)], synthesized under noncatalytic conditions (υ̅_CO_: 2026, 1932, 1914 cm^–1^).^28^ DFT calculations confirm this assignment, showing good agreement between calculated frequencies for an Mn-formate species (Mn–OCHO) with a deprotonated O–H group (**6-H** ^**+**^, Figure 3A,C, Table ST1). Therefore, these data together suggest that intramolecular proton transfer from one of the –OH groups to the Mn­(–I) center generates **2H–H** ^**+**^ which then reacts with CO_2_, forming the Mn–OCHO intermediate (**6-H** ^**+**^) with a rate constant (*k*_f_, _(**6‑H+**)_) of 0.70 ± 0.03 s^–1^ (Figure 4A, orange trace). This aligns with the following scheme, where the ″inverted″ kinetics for **2H–H** ^**+**^ (*k*_f_ < *k*_d_) explains its small signal amplitude and that the signal rises with *k*_d,(**6‑H+**)_ and decays with *k*_f,(**6‑H** ^+^)_: **2** ^**–**^ $\overset{k_{f}}{\to }$ **2H–H** ^**+**^ $\overset{k_{d}}{\to }$ **6-H** ^**+**^. The TOF for formate production, calculated from CPE, is 0.28 s^–1^ for **1-Br**,^12,29^ matching well with the observed *k*_f,(**6‑H+**)_. The 2024 cm^–1^ trace for **6H–H** ^**+**^ requires biexponential fitting, possibly due to CO accumulation in the solution, causing baseline shifts. We have also performed stopped-flow TRIR experiments with varying concentrations of CO_2,_ and the slope of the linear fit to the observed rate constant for **6-H** ^**+**^ formation vs. [CO_2_] gave a second -order rate constant of 9.5 ± 1.0 M^–1^ s^–1^ in anhydrous acetonitrile (Figure S7). Two additional vibrational bands appear at 1672 cm^–1^ and 1608 cm^–1^ (Figure S8). The 1672 cm^–1^ band likely corresponds to bicarbonate formation, a common side product in CO_2_-to-CO conversion. The 1608 cm^–1^ band grows at a similar rate as the 2024 cm^–1^ trace, suggesting it may represent the asymmetric C=O stretch of bound formate, though ligand-based stretching has also been reported near this wavenumber.^24^

### CO Formation Pathway

The TRIR spectra (Figure 3) also display the decay and formation of vibrational bands at around 2008, 2111, and 2138 cm^–1^. The 2008 cm^–1^ band was formed within the time-resolution of the experiment. This υ̅_CO_ stretch matches with the υ̅_CO_ of the [Mn­(mesbpy)­(CO)_3_COOH] species observed in previous FTIR-SEC experiments in CO_2_, as well as similar Re­(bpy-R)­(CO)_3_COOH complexes,^10,21,30^ and was therefore assigned to **4**. The calculated frequency for a proposed Mn­(I)-carboxylic acid species, **4** (Table ST1), also matches the observed frequency, suggesting that both hydroxyl groups remain protonated (Figure 5D). The lower frequency vibrations of **4** were masked by the strong absorption of **2** ^**–**^. A rapid decay of **4** was observed, with a rate constant of 2.6 ± 0.2 s^–1^ tracking the 2008 cm^–1^ trace (Figure 5A, red trace).

**Figure 5:**
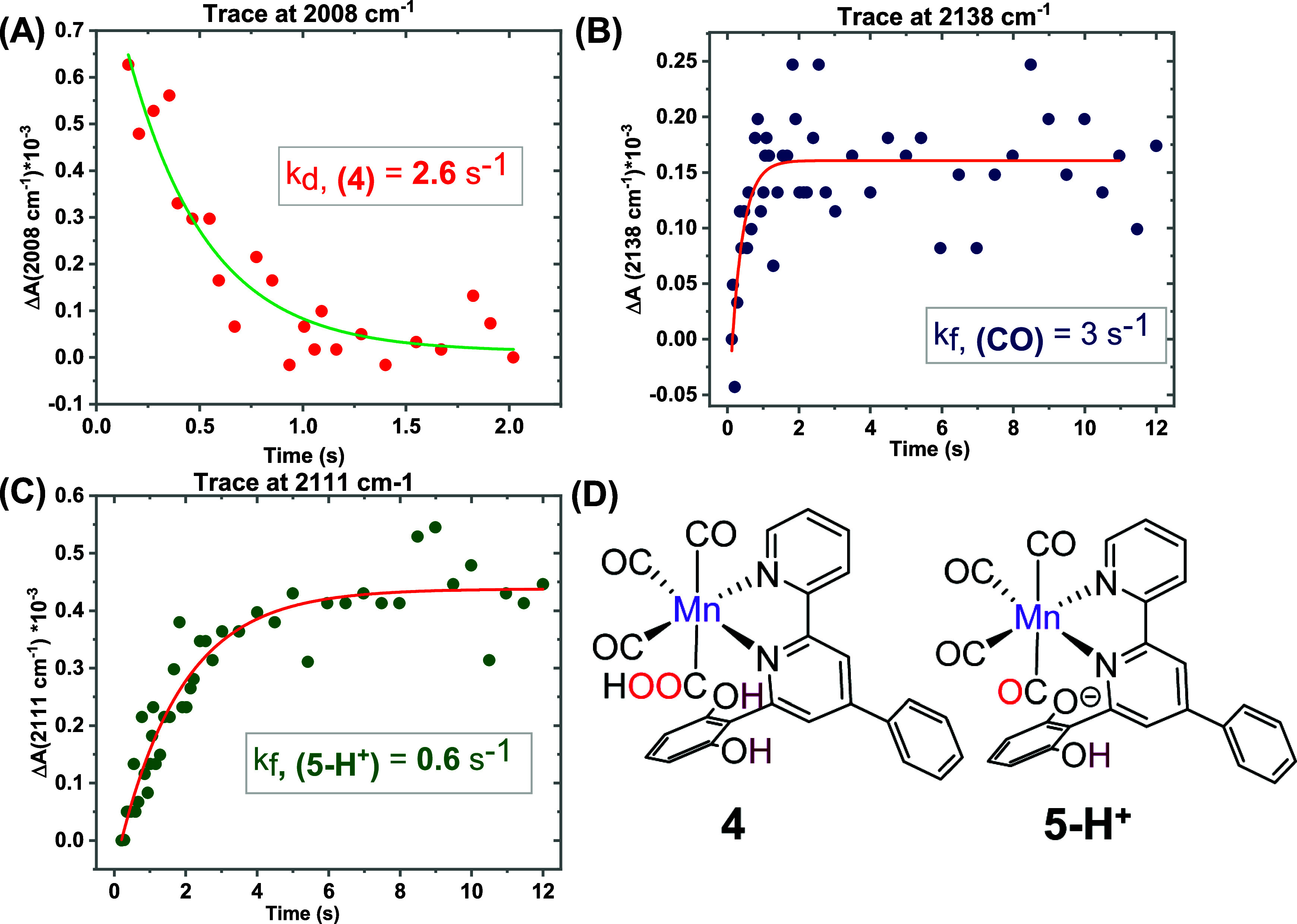
Kinetic traces of (A) **4** (red), (B) CO (blue), and (C) **5-H** ^**+**^ (green) during the reaction between 0.5 mM **5** and 2.6 mM CoCp_2_* in CO_2_-saturated anhydrous acetonitrile. (D) Chemical structures of the intermediates, **4**, and **5-H** ^**+**^. The rate constant values are mean values obtained from at least three individual experiments; standard deviations are given in the text.

The vibrational band at ∼2138 cm^–1^, corresponding to dissolved CO in acetonitrile,^23^ grows with a similar rate constant 3.0 ± 0.8 s^–1^, (Figure 5B, blue trace). This indicates that CO formation proceeds through the Mn-carboxylic acid intermediate (**4**), in agreement with the mechanistic scheme of Figure 1. The CO formation TOF from the CPE experiment with **1-Br** is 2.86 s^–1^,^12,29^ aligning well with the rate constant from our experiment, confirming that the latter mirrored e-CO_2_RR conditions.

The observation of υ̅_CO_ at 2111 cm^–1^ indicates a tetracarbonyl species^31^ that is red-shifted by 6 cm^–1^ compared to the 2117 cm^–1^ band of **5**, suggesting it corresponds to an oxidized tetracarbonyl species with a deprotonated O–H group in the second-sphere (**5-H** ^**+**^, Figure 5D). DFT-calculated vibrational frequencies support this hypothesis (Figure S9, Table ST1). The kinetic trace of **5-H** ^**+**^ (at 2111 cm^–1^) shows a slower formation rate (*k*_f, (**5‑H** ^+^)_ = 0.6 ± 0.07 s^–1^) compared to the decay or formation of **4** or CO, respectively. **5-H** ^**+**^ does not decay within the experimental time scale (Figure 5C), indicating that it does not contribute to CO production under these conditions.

The proposed mechanism for Mn­(bpy-R)­(CO)_3_X catalyzed e-CO_2_RR indicates that the Mn-catalyst can follow either a reduction-first or protonation-first pathway, depending on the applied potential and the p*K*_a_ of the proton source (Figure 1).^32,33^ At high cathodic potential with a weak proton source like water, the reduction-first pathway is preferred. Our DFT calculations show that the reduction potential for **4** → **4** ^**–**^ conversion is −1.42 V vs. SCE, lower than the CoCp_2_*^(+/0)^ potential in acetonitrile (−1.56 V vs SCE). Protonation of the reduced Mn-carboxylic acid (**4** ^**–**^) to form the reduced tetracarbonyl species (**2CO**) is exothermic (Δ*G* ^0^_reaction_ = −32.75 kcal/mol, Figure S11), suggesting that under our experimental conditions, the reduction-first pathway dominates, with **4** ^**–**^ and **2CO** being too short-lived to be detected. Attempts with singular-value decomposition (SVD) analysis of the multiwavenumber data failed to resolve additional spectral or temporal components. The slower decay of **2** ^**–**^, compared to the decay of **4** or the formation of CO, is counterintuitive, but can be rationalized by the fact that we are observing turnover conditions. We proposed that, as in the reaction mixture, both electrons (we have used an excess reductant) and protons (from the residual H_2_O, even in anhydrous acetonitrile, the water content can be around 0.01% (v/v),^34^ or ∼6 mM) are present, the CO formation cycle (though the reduction first mechanism) undergoes few turnovers, releases CO, and quickly regenerates the CO_2_-binding intermediate, **2** ^**–**^. That is proposed to be the main reason for not seeing any appreciable decay of the **2** ^**–**^ species during the earlier time. This type of behavior was observed in a previous IR-SEC experiment with the **1-Br** catalyst, where the absorbance of **2** ^**–**^ remains unaffected during IR-SEC, but the continuous growth of carbonate and bicarbonate bands was seen, which is often related to the catalytic CO production.^24^ However, the proton and reducing equivalents are depleted after a few cycles, causing the CO production cycle to stop. Subsequently, protons from one of the hydroxyl groups is used to protonate **4**, followed by the H_2_O release to yield **5-H** ^**+**^ with a rate constant of 0.6 s^–1^. This may explain the deactivation of **1-Br** within minutes during the CPE experiments in CO_2_-saturated anhydrous acetonitrile.^12^ After the cessation of the CO production cycle, the decay of **2** ^**–**^ was observed as an average of the slower formation of **5-H** ^**+**^ and the Mn–H species (and the corresponding Mn-formate intermediate). Since, at this effective reductant concentration, the release of HCO_2_^–^ from the Mn-formate species is difficult, the regeneration of **2** ^**–**^ is not possible through the formate pathway (Figures 1 and S11), resulting in the eventual decay of **2** ^**–**^ to Mn-formate species with a similar rate constant. DFT calculation also supports this hypothesis as the CO production cycle is thermodynamically much more favorable than the formate production pathway, as the latter requires the relocation of protons from the second-sphere hydroxyl groups to the metal center.

The experiment conducted under conditions similar to those in Figures 2 and 3, but without CO_2_, shows the bands corresponding to **2** ^**–**^ (with two intact OH groups), as observed in the presence of CO_2_ (Figure S12). However, the decay of **2** ^**–**^ (tracked at 1822 cm^–1^) under argon is nearly 10 times slower than the same in a CO_2_ atmosphere. We were unable to detect any signature of the Mn-hydride species at the TRIR time scale (23.5 s). We noted the growth of bands at 2012 cm^–1^, 1913 cm^–1^, and 1898 cm^–1^ with a rate constant similar to the decay of **2** ^**–**^. These bands closely matched a previously characterized intermediate species having deprotonated phenolic OH, in which one of the phenolates binds to the reduced metal center (**7**).^24^ Over time, species **7** decays, and a characteristic absorbance (albeit small) for the Mn-hydride intermediate (**2H–H** ^**+**^) is observed at υ̅_CO_ = 1988 cm^–1^ and 1975 cm^–1^ (sh). We propose that, in the presence of CO_2_, the reaction of species **2** ^**–**^ with CO_2_ is faster, consuming the reductant in the CO formation cycle. As a result, the deprotonation of the phenolic OH groups and/or the binding of the resultant phenolate to the metal center is avoided. However, in the absence of CO_2_, the excess reductant removes protons from the phenolic OHs, forming the corresponding phenolates. We propose that some Mn-hydride (**2H–H** ^**+**^) is generated by the relocation of one of the protons from the phenolic OH to the reduced metal center, which quickly reacts with protons in the solution to generate H_2_, as previously observed as a trace product from the CPE experiment with **1-Br** catalyst,^12^ and forms species **7** from the **2H–H** ^**+**^ species. At a later time scale, **2** ^**–**^ decays completely, leaving an appreciable concentration of Mn-hydride species (**2H–H** ^**+**^). These results clearly show the competition between the productive CO_2_ reduction and the unproductive ligand coordination leading to the deactivation of the catalyst.

### TRIR Experiments in the Presence of 5% H_2_O

Adding 5% H_2_O as a proton source during e-CO_2_RR increased the electrocatalytic current for **1-Br**, as seen with other Mn catalysts.^35^ However, product selectivity shifted, with FY_CO_ rising to 90% and FY_HCOOH_ dropping to 4%. This unusual shift led us to further investigation. A similar electrocatalytic current increase was observed for **5** with 5% H_2_O (Figure S13). Therefore, we performed a similar stopped-flow TRIR study (vide supra) in the presence of 5% H_2_O. The TRIR data (Figure 6A) show the presence of **2** ^**–**^ from the start, decaying quickly with a rate constant of 8.5(±0.4) s^–1^ (Figure 6C, purple trace). The υ̅_CO_ band at 1988 cm^–1^ for **2H–H** ^**+**^ was also present initially, and its formation kinetics could not be captured, although its decay was observed with a rate constant of 9.9 (±0.9) s^–1^ (Figure 6D). The Mn–OCHO intermediate, **6-H** ^**+**^ accumulates with a rate constant of 9.0 (±0.3) s^–1^ (Figure 6C). Additionally, the second-order rate constant for the formation of **6-H** ^**+**^ species was calculated to be 110 ± 10 M^–1^ s^–1^, from the slope of the linear fit to the observed rate constant at five different CO_2_ concentrations (Figure S14).

**Figure 6:**
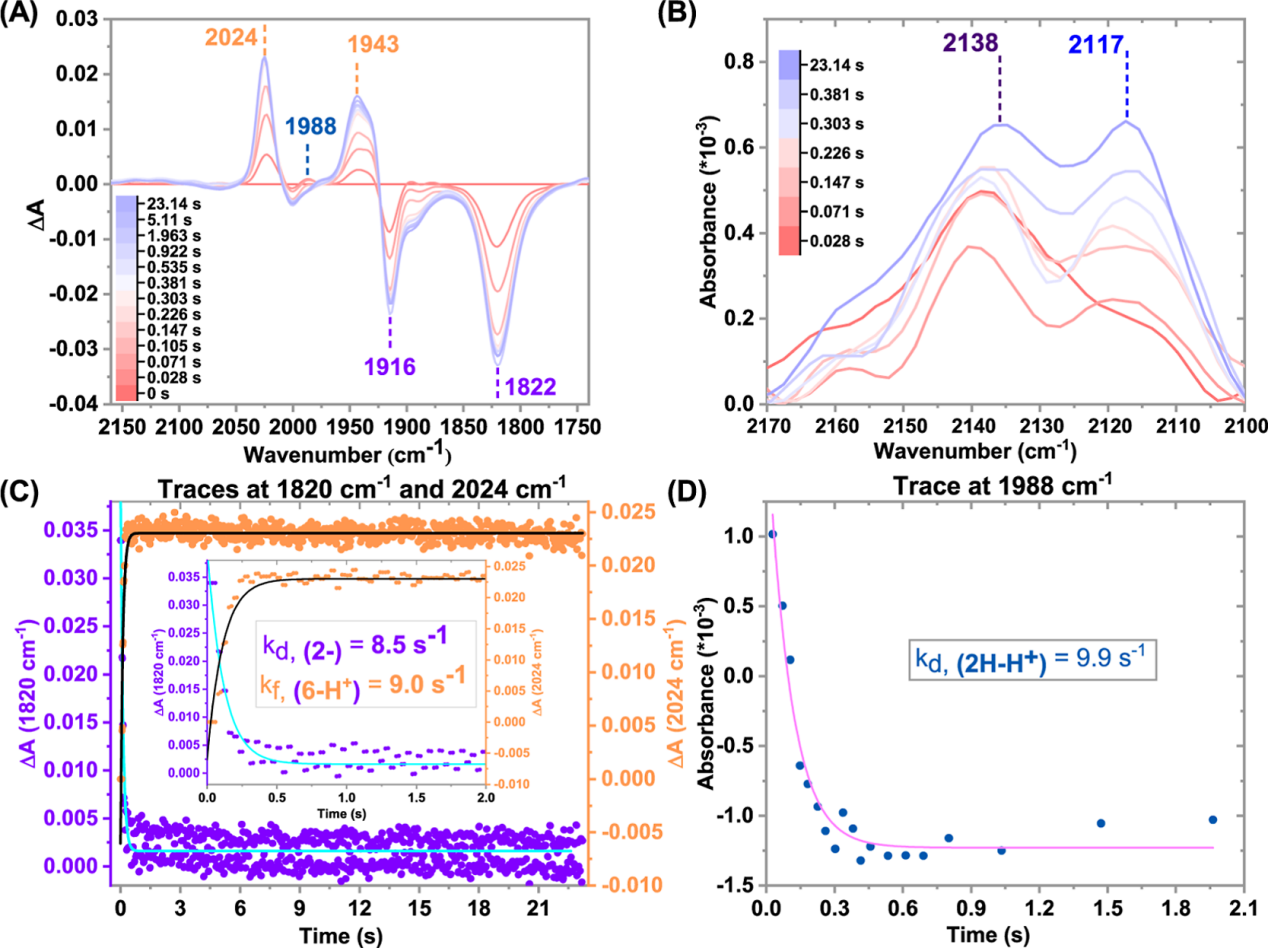
(A) TRIR difference spectra of 0.5 mM of complex **5** in acetonitrile solution showing the spectral changes upon adding 2.6 mM CoCp_2_* in CO_2_-saturated acetonitrile containing 5% H_2_O. (B) Zoomed-in section of (A). Kinetic traces of (C) **2** ^**–**^ (purple), **6-H** ^**+**^ (orange), and (D) **2H–H** ^**+**^ (turquoise). In these figures, the decay of the species present at the initial time (within the time resolution of the instrument) and the formation of new species are represented as negative bands and positive bands, respectively. The rate constant values are mean values obtained from at least three individual experiments, standard deviations are given in the text.

Interestingly, even with 5% H_2_O, protons from the second-sphere hydroxyl group are essential for forming the Mn-hydride complex as the υ̅_CO_ band at 1988 cm^–1^ corresponds to the **2H–H** ^**+**^, i.e., one of the hydroxyl groups loses a proton. Then CO_2_ inserts into this Mn-hydride bond forming the Mn–OCHO intermediate­(**6-H** ^**+**^), a critical intermediate in the HCOOH formation pathway. The relative absorbance ratios of the 2024 cm^–1^ (**6-H** ^**+**^) and 1822 cm^–1^ (**2** ^**–**^) bands remain almost unchanged (Figures 2 and 5A) when shifting from dry to wet (5% H_2_O) acetonitrile, indicating a similar product distribution under both conditions. This contrasts with previously reported FY values in wet acetonitrile (vide supra).^12,24^ To investigate further, we conducted a CPE experiment with **5** at −1.9 V vs. Fc^+^/^0^ for 2 h in CO_2_-saturated wet acetonitrile (5% H_2_O). A slight shift in product selectivity was observed, with FY_CO_ increased to ∼72% and FY_HCOOH_ decreased to ∼13% (Figure S15), consistent with the TRIR data.

The signatures of neither Mn–COOH species nor other intermediates were observed in the CO formation pathway, except for a broad band at 2138–2140 cm^–1^, indicating CO in acetonitrile. This indicates that the steps of the CO formation pathway are too rapid to accumulate other intermediates than **2** ^**–**^ (i.e., **2** ^**–**^ → **4** is rate limiting), and water, not acidic enough to protonate **2** ^**–**^, accelerates the CO pathway, aligning with the observed change in selectivity. As the concentration of CoCp_2_* decreased, **5** (having two intact –OH groups) gradually accumulated (Figure 6B), indicating that in the presence of 5% H_2_O, the protonation and subsequent dehydration of **4** do not require the internal protons.

Using 5% D_2_O as the external proton source, we observed the signature of the **2H–H** ^**+**^ (υ̅_CO_ at 1988 cm^–1^) intermediate (Mn-hydride intermediate) from the initial time. **2H–H** ^**+**^ species then decays to the corresponding Mn-formate species (**6-H** ^**+**^), displaying clear υ̅_CO_ at 2024 cm^–1^ and 1943 cm^–1^ (Figure S16). This observation aligns with the same experiment using 5% H_2_O as the proton source, i.e., the internal proton from one of the –OH groups required to form the corresponding Mn-hydride intermediate toward formate production. This supports our assignment about the protonation state of the intermediates. However, we observed a decrease in the rate constant compared to the same in the 5% H_2_O condition, bearing a KIE = 1.2. The slightly lower rate constant in the D_2_O case might be due to the lower tunneling probability of heavy deuterium from one of the -OD groups to the Mn­(–I) center. Similar to the 5% H_2_O case, the intermediates in the CO formation pathways are too transient to be detected here. Additionally, as the effective concentration of CoCp_2_* decreases due to the turnover of the CO pathway, species **5** (which has two intact –OH groups) gradually accumulates (Figure S16).

In principle, adding H_2_O (or D_2_O) should accelerate only the CO formation pathway, as they are not acidic enough to form the Mn-hydride complex en route toward formate production. Therefore, the rate constants for the Mn-hydride or the Mn-formate species are not expected to be affected upon H_2_O (or D_2_O) addition, as the relocation of the internal proton from one of the –OH groups to the reduced metal center should be the same in the presence or absence of them. On the contrary, our results show an increase in the formation rate constant of the Mn-formate complex, **6-H** ^**+**^, and the decay of Mn-hydride species, **2H–H** ^**+**^, upon H_2_O (or D_2_O) addition (Figures 6 and S16). To inquire about the possible explanation, we modeled **2** ^**–**^ in the presence of one nearby H_2_O molecule. The optimized geometry shows that the presence of only one H_2_O molecule reduces the distance between the phenolic-O­(H) to the Mn­(–I) center by 0.11 Å (Figure 7). That might help in faster relocation of the phenolic proton to the Mn­(–I) center, making the acceleration of the formate production rate in the presence of H_2_O.

**Figure 7:**
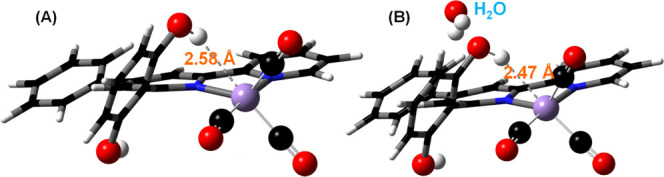
DFT optimized (b3lyp/def2-tzvp with GD3BJ and CPCM/MeCN) geometry of (A) [Mn­(dhbpy)­(CO)_3_]^−^ species (**2** ^**–**^) and (B) [Mn­(dhbpy)­(CO)_3_]^−^ species (**2** ^**–**^) in the presence of a nearby H_2_O molecule. The H-bonding distance from one of the pendant O–Hs to the reduced Mn­(–I) center is shown here.

## Conclusion

In conclusion, we provide the first time-resolved spectroscopic evidence of bifurcation pathways for CO and HCOOH formation in real time during e-CO_2_RR conditions, catalyzed by a Mn bipyridine tricarbonyl complex that features second-sphere hydroxyl functional groups. Coupling stopped-flow mixing with TRIR spectroscopy, we identified, for the first time, the key intermediates in the CO and HCOOH bifurcation pathway and tracked their kinetics in operando. The rate constants obtained align with TOF reported in previous electrochemical studies, demonstrating the ability of our experiment to replicate e-CO_2_RR conditions. The stopped-flow TRIR data under argon in anhydrous acetonitrile highlighted that the productive CO_2_ reduction rate is much faster than the unproductive phenolate coordination. Therefore, the latter might not be the primary reason for catalyst degradation during e-CO_2_RR. Our findings also reveal that HCOOH production proceeds via proton transfer from a hydroxyl group to the Mn­(–I) center, forming a Mn-hydride intermediate and corresponding metal-formate species. However, the inability of the resulting phenolate to rebind protons, even in the presence of 5% H_2_O, leads to rapid catalyst degradation. The reprotonation in that case by water is highly desired, as the use of a highly acidic proton source also compromises the selectivity, and might shift the selectivity more toward H_2_. Therefore, amine-based functional groups are highly sought in this regard as their deprotonated forms are basic and could be reprotonated in wet (5% H_2_O) acetonitrile. This efficient reprotonation will help in improving catalyst stability under operation without sacrificing the selectivity.

## Supplementary Material



## Notes

The Supporting Information file (PDF) includes detailed experimental procedures, synthesis, and characterization of the complex, electrochemical data, e-CO_2_RR product characterization data using GC-TCD and ion chromatography, calculated vibrational frequencies, a proposed catalytic cycle including thermochemical data, and the Cartesian coordinates of the DFT-optimized structures (PDF)
