# Supplementary material for: Unraveling Bifurcating Pathways for CO and HCOOH Formation: Insights from Stopped-Flow FTIR Spectroscopy of a Second-Sphere Modified Mn Catalyst
Source: J Am Chem Soc. 2025 Jun 18;147(26):22697–704. doi: 10.1021/jacs.5c04274 (PMC12232292; doi:10.1021/jacs.5c04274)
Supplement: Supplementary file 1 [file ja5c04274_si_001.pdf]

Supporting Information for

Unraveling Bifurcating Pathways for CO and HCOOH Formation: Insights from Stopped-Flow FTIR Spectroscopy of a Second-Sphere Modified Mn Catalyst

Samir Chattopadhyay,[1] Sudip Barman,[2] Reiner Lomoth,[1] Leif Hammarström[1]*

[1]Department of Chemistry - Ångström Laboratories, Uppsala University, Uppsala SE 75120, Sweden

[2]School of Chemical Sciences, Indian Association for the Cultivation of Science, 2A Raja SC Mullick Road, Kolkata, West Bengal 700032, India

Email: leif.hammarstrom@kemi.uu.se

Table of Contents

1. General information………………………………………………...………………..S3-S9

2. Figure S1: FTIR spectrum of [Mn(dhbpy)(CO)$_4$]SbF$_6$ (**5**)…………………………….…S10

3. Figure S2 and S3: Product characterizations using GC-TCD (for gaseous products) and ion-chromatography (for liquid products) obtained after control potential electrolysis at -1.9 V vs Fc$^{+/0}$ under CO$_2$.……………………..…………………………….…………………S10-S11

4. Figure S4 and S5: Calibration curves for GC-TCD and ion-chromatography.……….S11-S12

5. Figure S6: Schematic representation of the mixing sequence used in the stopped-flow TRIR experiments ……………………………………………………………………. …..S12

6. Figure S7: The formation rate constant of Mn-formate vs. [CO$_2$] in acetonitrile………..S13

7. Figure S8: Kinetic traces at 1608 cm^{-1} and 1672 cm^{-1}……………………..….…………S13

8.Figure S9: Correlation between the experimental & DFT calculated $\bar{\upsilon}_{CO}$ bands…………...S14

9. Figure S10: DFT optimized geometry of [Mn(dhbpy)(CO)$_3$]$^-$ showing H-bonding……...S14

10. Figure ST1: Experimental and DFT calculated $\bar{\upsilon}_{CO}$ bands of the species…………..…….S15

11. Figure S11: Proposed catalytic cycle of CO$_2$ to CO and HCOOH production with thermodynamic…………………………………………………………………...S16

11. Figure S12: TRIR spectra and kinetics of various intermediates under argon…………....S17

12. Figure S13: Cyclic voltammogram of complex **5** under CO$_2$ in the absence and presence of 5% H$_2$O……..………………………………………………………………....……S18

13. Figure S14: The formation rate constant of Mn-formate vs. [CO$_2$] in wet acetonitrile..-S19

14. Figure S15: Product analysis after CPE under CO$_2$ in the presence of 5% H$_2$O …………………………………..……………………………………………......S19

15. Figure S16: TRIR spectra in the presence of 5% D$_2$O under CO$_2$ atmosphere…………....S20

16. Cartesian coordinates of the optimized geometries……………………………....S20-S31

17. References……………………………………………………….…………………S31-S32

1. General Considerations:

1.1. Materials and Instrumentations: All chemicals used are commercially available, of the highest purity grade, and used without further purification. Benzaldehyde, 2-Acetylpyridine, iodine, potassium hydroxide, pyridine, ammonium acetate, pyridine hydrochloride, $Mn(CO)_5Br$, decamethyl cobaltocene ($CoCp_2$*), methanol, and silver hexafluoroantimonate ($AgSbF_6$) were purchased from Sigma-Aldrich. 2,6-dimethoxyacetophenone was purchased from BLDPharm-Germany. Deoxygenated dichloromethane (DCM), pentane, and acetonitrile (HPLC grade, purchased from VWR) were collected from an Inert® solvent purification system and stored over activated 4 Å molecular sieves for at least 48 hrs. before use. Acetone-d^6 and acetonitrile-d^3 were purchased from Sigma-Aldrich. The gas-tight® Hamilton® syringes used in the stopped-flow IR experiments were purchased from Sigma-Aldrich.

All anaerobic experiments (synthesis and sample preparation for the stopped-flow experiments) were performed in an MBraun glovebox (argon-filled). All NMR spectra are recorded at 298 K using a JEOL Eclipse+ 400 MHz spectrometer, with ^1H and ^{13}C NMR experiments run at 400 MHz and 101 MHz respectively. The corresponding chemical shifts are reported relative to TMS (chemical shift = 0) and referenced against solvent residual peaks.

All cyclic voltammograms were collected on a Metrohm Autolab potentiostat (PGSTAT 302) with Nova 2.1.4 software in a one-compartment three-electrode configuration with glassy carbon electrode, platinum wire, and non-aqueous $Ag/AgNO_3$ electrode as working, counter, and reference electrode respectively. The reference electrode was calibrated using the $Fc^{+/0}$ redox couple before and after each set of experiments and the final cyclic voltammograms are reported with respect to the $Fc^{+/0}$ redox couple. Controlled potential electrolysis experiments were performed on a CH instruments electrochemical analyzer (CHI700E). An Agilent GC instrument (model no.: 7890B, G3440B, serial no. CN14333203) fitted with a TCD and mass detector was used to analyze the headspace gas mixture during product analysis. The liquid

product, formate, was determined using a Metrohm ion chromatography instrument (serial: 0022.2486) fitted with an anion column (6.1006.530).

1.2. Synthesis:

1.2.1. Synthesis of the dhbpy ligand, 2-(4-phenyl-2,2'-bipyridin-6-yl)benzene-1,3-diol: The dhbpy ligand was synthesized following the synthetic protocol reported by Nervi, Gobetto, and co-workers.[1] ^{1}H NMR (DMSO-d^6, 400 MHz): δ (ppm) = 12.15 (s, 2H, OH) 8.76 (m, 2H), 8.48 (s, 1H), 8.20 (d, 1H), 8.02 (t, 1H), 7.83 (d, 2H), 7.56 (m, 2H), 7.51 (m, 2H), 7.08 (t, 1H), 6.46 (d, 2H). ^{13}C NMR (in DMSO-d^6, 101 MHz): δ (ppm) = 159.2, 156.6, 154.5, 153.4, 150.4, 150.3, 138.3, 131.4, 130.1, 129.9, 127.6, 125.2, 123.5, 121.1, 116.9, 109.3, 108.1.

*1.2.2. Synthesis of Mn(dhbpy)$(CO)_3$Br (**1-Br**):* Following the same report,[1] **1-Br** was synthesized. ^{1}H NMR (Acetone-d^6, 400 MHz): δ (ppm) = 9.30 (d, 1H), 8.81 (m, 2H), 8.73 (s, 1H), 8.21 (t, 1H), 8.03 (d, 2H), 7.85 (s, 1H), 7.71 (t, 1H), 7.55 (m, 3H), 7.22 (m, 2H), 6.64 (d, 1H), 6.57 (d, 1H). FTIR (ATR, $\bar{\upsilon}_{CO}$, cm^{-1}): 3322, 2018, 1929, 1898.

*1.2.3. Synthesis of [Mn(dhbpy)$(CO)_4$]SbF_6 (**5**):* Compound **5** was synthesized following the protocol reported by Bocarsly and coworkers for the synthesis of [Mn(bpy)$(CO)_4$]SbF_6.[2] Typically, 500 mg (0.89 mmol) of **1-Br** and 615 mg (1.79 mmol) of $AgSbF_6$ were dissolved in dry, degassed dichloromethane (DCM) under a CO atmosphere. The reaction was run for 1 hour in the dark under continuous CO purging. The reaction mixture was then filtered to remove AgBr, and the filtrate was concentrated in vacuo. The resulting solution was kept in the dark at -20°C overnight, leading to the precipitation of **5**. The product was filtered and dried under a high vacuum, yielding approximately 40% of compound **5**. ^{1}H NMR (Acetone-d^6, 400 MHz): δ (ppm) = 9.25 (d, J = 4.7 Hz, 1H), 9.08 – 8.95 (m, 2H), 8.44 (td, J = 7.9, 1.5 Hz, 1H), 8.13 (d, J = 2.1 Hz, 1H), 8.12 – 8.08 (m, 2H), 7.92 (ddd, J = 7.2, 5.7, 1.4 Hz, 1H), 7.61 (d, J = 1.3 Hz, 1H), 7.60 (d, J = 2.4 Hz, 2H), 7.28 (t, J = 8.3 Hz, 1H), 6.65 (d, J = 8.3 Hz, 2H). FTIR (in acetonitrile) $\bar{\upsilon}_{CO}$: 2116 cm^{-1}, 2042 cm^{-1}, 2015 cm^{-1}, 1974 cm^{-1}. HRMS (ESI-NS, CH_3CN/H_2O) m/z: $[M]^+$: Calculated for $C_{26}H_{16}N_2O_6Mn$: 507.04, found 507.03844, $[Mn\text{-}CO]^+$: calculated for $C_{25}H_{16}N_2O_5$: 479.04, found: 479.04342.

1.3. Electrochemical Studies:

1.3.1. Cyclic voltammetry

All CV experiments were performed using 1 mM [Mn(dhbpy)$(CO)_4$]SbF_6 (**5**) and 100 mM tetra butyl ammonium hexafluorophosphate (as supporting electrolyte) in dry and degassed

acetonitrile. The solutions were purged by bubbling argon or CO_2 before recording the CV as required. In these experiments, a glassy carbon electrode, platinum wire, and non-aqueous Ag/$AgNO_3$ electrodes were used as working, counter, and reference electrodes respectively. The potential of the reference electrode was calibrated using $Fc^{+/0}$ redox couple before and after each experiment and reported with respect to $Fc^{+/0}$ redox couple.

1.3.2. Control potential electrolysis (CPE) and product analysis:

1 mM **5** and 100 mM tetra butyl ammonium perchlorate were dissolved in 5 mL dry and degassed acetonitrile. This solution was poured into a sealed four-necked two-compartment electrochemical cell and purged with CO_2 for 1 hr. until the solution gets saturated. The CPE experiments were performed on a CHI 700E bi-potentiostat for 2 hrs. at the mentioned potentials using a glassy carbon plate (area: 1cm^2), a platinum wire attached with a glass-frit containing an acetonitrile solution of tetra butyl ammonium perchlorate, and a standard double-junction aqueous Ag/AgCl as the working, counter, and reference electrodes respectively.

The gaseous products were collected into a burette by the vertical displacement of water. The volume of water displaced during the CPE experiment corresponds to the amount of gas evolved. The gas was collected from the head-space for analysis using an Agilent instrument (model: 7890B) fitted with a TCD detector (G3440B).

Any liquid products, obtained after the CPE experiment, was extracted using $CHCl_3$-water. The resulting aqueous layer was filtered and injected into a Metrohm ion-chromatography instrument fitted with an anion column. A solution of 3.2 mmol/L sodium carbonate and 1.0 mmol/L sodium hydrogen carbonate were used as the eluents. The formate detection was performed maintaining a flow-rate of 0.4 mL/min.

1.3.3. Faradaic yield calculations

1.3.3.1. Carbon monoxide (CO):

The calibration process of the GC-TCD instrument was done by injecting varying volumes of an H_2:CH_4 (1:1) gas mixture, varying from 250 µL to 2000 µL. The relative sensitivity of each specific gas (H_2, CO for this work) in the TCD detector was then determined by analyzing the slope of the TCD counts versus the injection volumes plot. The slope of the plot for both CO and H_2 (*Figure S5*) indicates that the sensitivity of CO is 40.68 times that of H_2, calculated as (276033/6785). The sensitivity ratio was then utilized to calculate the volume of each gas during the determination of Faradaic yields. The CPE experiment performed with 1 mM **5** in 5 mL

acetonitrile under CO_2 at -1.9 V *vs.* $Fc^{+/0}$ for 2 hrs. shows only CO as the gaseous product upon consuming 10.65 C charge. The volume of the CO was estimated from the water replacement in an inverted burette. The total volume of CO in the headspace of the inverted burette is 0.84 mL. Amount of CO produced = 0.84/22400 = 37.5×10^{-6} mol. FY of CO = ($2\times37.5\times10^{-6}$ ×96500)/10.65 = 67.9%.

The CPE experiment performed under similar conditions but in the presence of 5% H_2O consumed 18.1 Columb charge and shows CO as the only gaseous product. The total volume of the gas in the headspace is 1.5 mL. Amount of CO produced = 1.5/22400 = 6.69×10^{-5} moles. Therefore, FY of CO = 71.3%.

1.*3.3.2. Formate ($HCOO^-$):*

CPE at -1.9 V (*vs.* $Fc^{+/0}$) for 2 hrs. with 1 mM of 5 in anhydrous acetonitrile (5mL) shows the formation of formate as a liquid product detected through ion exchange chromatography (IC). The concentration of formate produced in the sample was found to be 84.998 ppm using the calibration curve (*Figure S6*). The total volume of the extract (vide supra) was 5 mL.

Reaction: $CO_2 + 2e^- + 2H^+ \rightarrow HCOOH$

FY_{HCOOH} (%) = (nFC/Q)×100

Where, n = 2 (no. of electrons required for $CO_2 \rightarrow HCOO^-$), F = Faraday constant, C = concentration of formate in moles (9.44×10^{-6} mol), Q = charge consumed during CPE (10.65 C).

Therefore, FY_{HCOOH} (%) = 17.1%.

Similarly, the amount of formate produced during CPE under similar conditions but in the presence of 5% H_2O is 12.35×10^{-6} mol. Total charge consumed 18.1 C.

Therefore, FY_{HCOOH} (%) = 13.17%

1.3.4. TOF calculation for CO and HCOOH formation:

Turnover frequency (TOF) values for CO and HCOOH formation were calculated following the Costentin and Savéant method.[3, 4]

$$\frac{i}{FA} = \frac{\sqrt{D}k_{cat}[C]_p^0}{1 + \exp[\frac{F}{RT}(E - E^0_{CO2 \cdot\cdot CO,S,AH})]} \quad \text{....................(1)}$$

$$k_{cat} = \frac{i^2(1+\exp[\frac{F}{RT}(E-E^0_{CO2 \therefore CO,S,AH})]^2}{F^2A^2D([C]^0_p)^2} \quad \text{......................(2)}$$

$$TOF = \frac{k_{cat}}{1+\exp[\frac{F}{RT}(E-E^0_{CO2 \therefore CO,S,AH})]} \quad \text{.....................(3)}$$

$$\approx k_{cat} \text{ when } (E - E^0_{cat}) < 0.1 \quad \text{..............................(4)}$$

Here, i = current observed during control potential electrolysis (CPE), F = Faraday's constant, A = surface area of the electrode, k_{cat} = rate constant of the catalytic reaction, D = diffusion coefficient of the molecule in the solution, R = universal gas constant, T = temperature, $[C]_p^0$ = concentration of the catalyst in the solution, E = applied potential during CPE, E_{cat}^0 = potential at which catalysis occurs, $E^0_{CO2/CO,s, AH}$ = standard potential for the conversion of CO_2 to CO in a solvent, s, (-3.1 V vs. SCE in acetonitrile). This value was not significant if the applied potential in the CPE experiment is much less than -3.1 V *vs.* SCE. Therefore, the term, $\exp[\frac{F}{RT}(E - E^0_{CO2 \therefore CO,S,AH}) \approx 0$ leading to the numerator in the equation 2 to be i^2.

For Mn(dhbpy)$(CO)_3$Br complex, we recalculated the TOF for CO and HCOOH formation using its previous reported values of the variables.[1]

i = 0.007336 A

η_{CO} = 70%, i_{CO} = (0.007336 × 0.7) A = 0.0051352 A

η_{HCOOH} = 22%, i_{HCOOH} = (0.007336 × 0.22) A = 0.001614 A

D = 1×10^{-5} cm^2s^{-1} [3, 5]

A =12.35 cm^2

C_{cat} = 7.703 × 10^{-7} mol cm^{-3}

TOF_{CO} = 2.84 s^{-1}

TOF_{HCOOH} = 0.28 s^{-1}

1.4. Stopped-flow FTIR experiments: A BioLogic SFM-3000 stopped-flow mixing instrument, having three syringes, was utilized to perform the rapid mixing at the FT-IR observation cell. The stopped-flow mixing system was connected to the FT-IR observation head with a 45 cm-long umbilical link (*from BioLogic*) that allows for conformational flexibility. **5** and CO_2 (or CO_2 in the presence of 5% H_2O) were mixed before traveling through the umbilical link and finally mixed with $CoCp_2^*$ at the observation cell after traveling the solutions separately

through the umbilical link. The observation head comprised both inlet (from the mixing instrument) and outlet (towards the waste cell) ports allowing the sample to flow through the small-volume observation chamber created by a 500 µm PTFE spacer placed between two CaF_2 windows. All of the stopped-flow FTIR spectroscopic measurements were performed at 25^0C. The total flow rate in all of the stopped-flow experiments was 8 mL/min. The stopped-flow mixing instrument, FT-IR observation cell, and waste port were flushed with nitrogen for at least 20 minutes before each set of experiments. In all of the experiments, syringes 1, 2, and 3 were filled with a saturated solution of CO_2 in acetonitrile (or CO_2 in MeCN having 5% H_2O), **5** in MeCN, and $CoCp_2$* in MeCN respectively (*Figure S7*).

The solutions of [Mn(dhbpy)$(CO)_4$]SbF_6 (**5**), $CoCp_2$* in acetonitrile, and saturated solutions of CO_2 in acetonitrile (either in the absence/presence of 5% H_2O) at the mentioned concentrations were prepared in an argon-filled glove box. Then, the solutions were filled into gas-tight® Hamilton® syringes and transferred from the glovebox to the stopped-flow mixing instrument. A three-way luer stopcock (from *Fisher Scientific*) was used to connect the Hamilton® syringes to the loading ports of the stopped-flow instrument. Then the whole system was purged at least six times with nitrogen to remove any O_2 that may enter through the loading ports during the installation of the Hamilton® syringes and kept under a nitrogen atmosphere throughout the experiment.

Rapid scan infrared spectra in absorbance mode were collected using a Bruker Vertex 70v instrument equipped with a liquid-N_2 cooled MCT detector. The interferometer compartment was kept under vacuum and the sample compartment was kept under a constant N_2 flow during the experiments. The BioLogic stopped-flow instrument was interfaced directly with the Bruker 70v via BNC cables. The full IR spectrum was collected in each 19 ms interval with 8 cm^{-1} resolutions. Flushing the FT-IR observation cell with CO_2-saturated MeCN from syringe 1 was used to record the background solvent spectra before each set of experiments. During the experiments with 5% H_2O, the 1st spectrum in the TRIR data was taken as the background. All of the rapid scan FTIR data were collected and handled with OPUS 8.2 software.

1.5. All of the other IR data were collected using the same instrument (liquid N_2-cooled MCT as the detector) with 4 cm^{-1} resolutions. A liquid transmission cell (250 µm optical path length) was used for the same.

1.6. FTIR kinetic data analysis: Origin 2019 software was used to analyze the FTIR data and prepare the final figures. There is more than one characteristic vibrational band present for the

initial complex (**5**), and the other intermediates species generated during the stopped-flow experiments. In this study, we focused on the higher vibrational bands of the intermediates for characterizations and kinetic analysis as the lower vibrational bands often overlap with those of other intermediates. However, for $[Mn(dhbpy)(CO)_3]^-$ intermediate, $\mathbf{2}^-$, we use the lower vibrational band at 1820 cm^{-1} for kinetic analysis as the other band merges with $[Mn(dhbpy\text{-}H^+)(CO)_3OCHO]$ intermediate, **6-H**$^+$. The kinetic traces were fitted using one or two single exponential functions using the Origin software's curve fitting utility.

1.8. Density Functional Theory (DFT) calculations: All geometry optimizations with Gaussian 09 Rev. E.01(*Gaussian/09.E.01-avx-nsc1-bdist*) software package.[6] Solvent effects were included in the calculations using a conductor-like conductor model (CPCM) with acetonitrile as the solvent.[7, 8] Geometry optimizations were performed using B3LYP functional and Def2-TZVP basis sets.[9-13] The D3 version of Grimme's dispersion function adopting the Becke-Johnson damping scheme was utilized during the calculations.[14, 15] Frequency calculations were also performed on each optimized geometry with the same functional and basis sets to ensure that it was a minimum of the potential energy surface. Gibbs free energies were extracted using the thermal correction for enthalpy and entropy at 298K to the electronic energies.[16] The slope of the linear fitting (0.96977) of the correlation between the experimental and DFT calculated vibrational bands of **5** (*Figure S10*) was used as the scaling factor for all computed harmonic frequencies shown in this work.

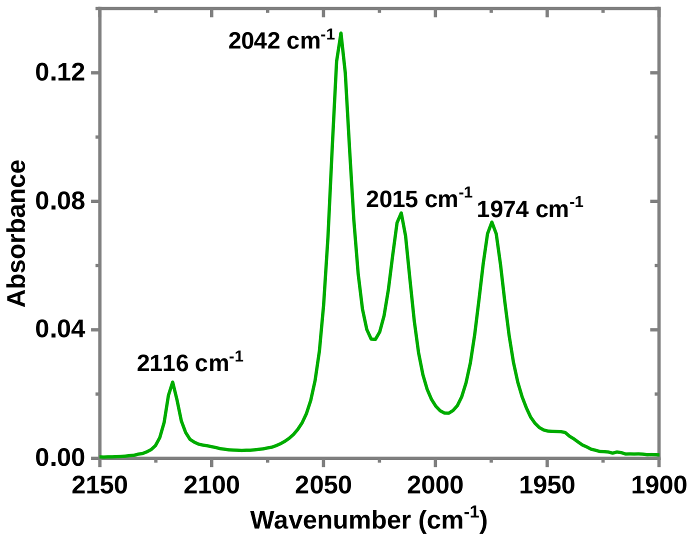

Figure S1: FTIR spectra of **5** in acetonitrile.

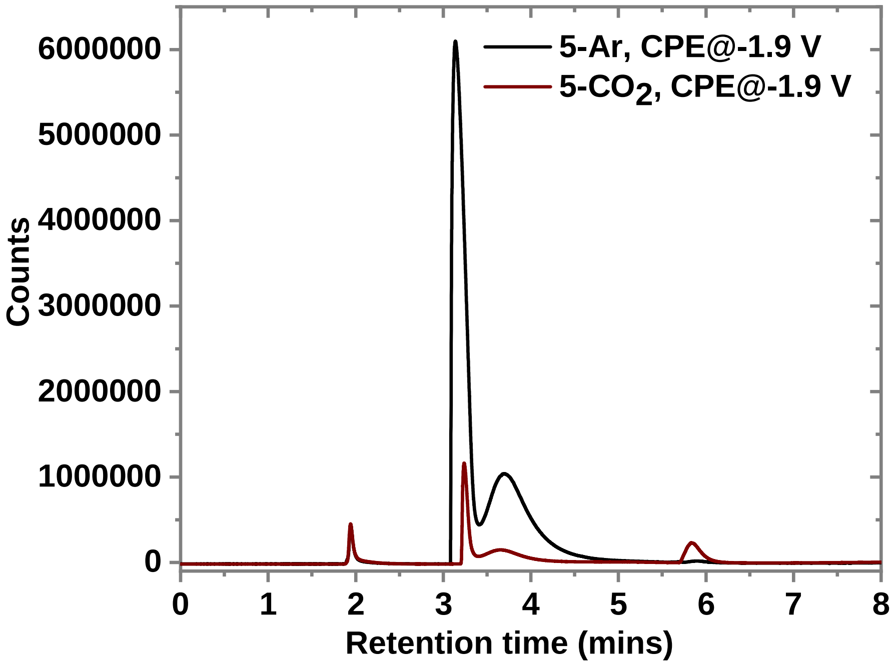

Figure S2: GC-TCD data of the gaseous product obtained after CPE at -1.9 V (*vs.* Fc/Fc$^+$) for 2 hrs. in the presence of 1 mM **5** under argon (black) and CO_2 (brown) atmosphere.

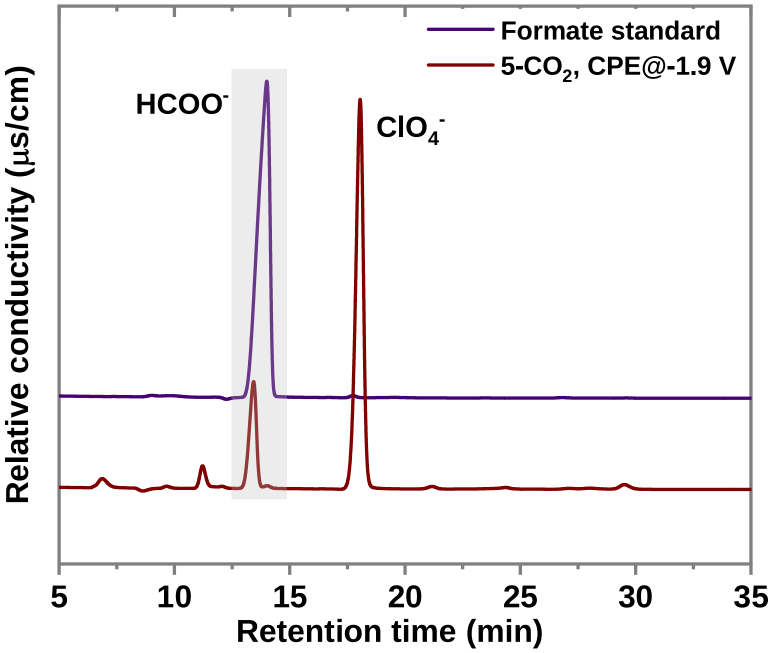

Figure S3: Product analysis by ion-chromatography: chromatogram of pure $HCOO^-$ is in purple, brown chromatogram shows the data collected after CPE at -1.9 V (*vs.* Fc/Fc^+) for 2 hrs. in the presence of 1 mM **5** under CO_2 atmosphere.

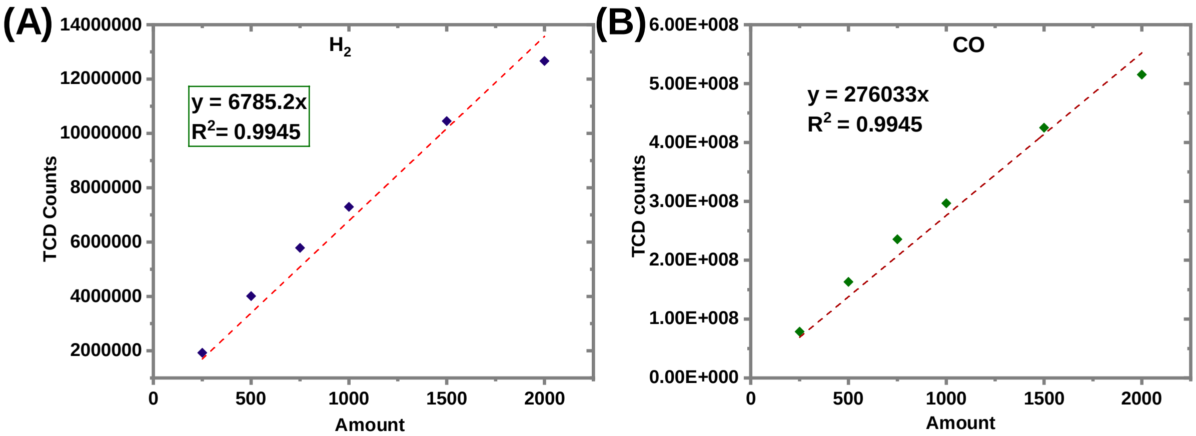

Figure S4: The calibration curve for H_2 (**A**) and CO (**B**) obtained by injecting a gas mixture (H_2: CO: CH_4: C_2H_6 = 1:1:1:1) by varying the volume of the gas ranging from 250 µL to 2000 µL. This calibration curve was used to ascertain the relative sensitivities of each gas in the TCD detector.

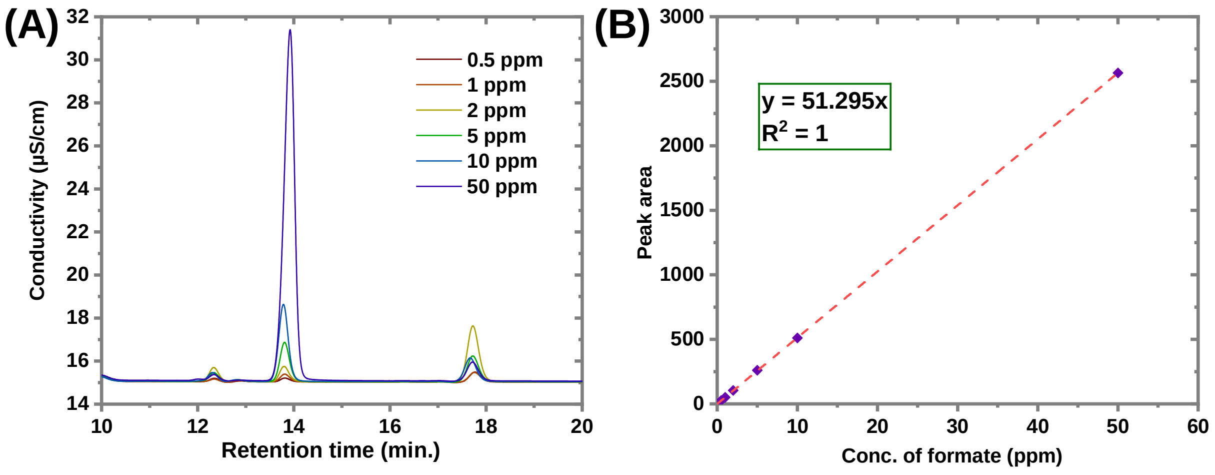

Figure S5: (**A**) Ion chromatograms of ammonium formate at different concentrations. (**B**) calibration curve obtained from the peak area of the formate peak.

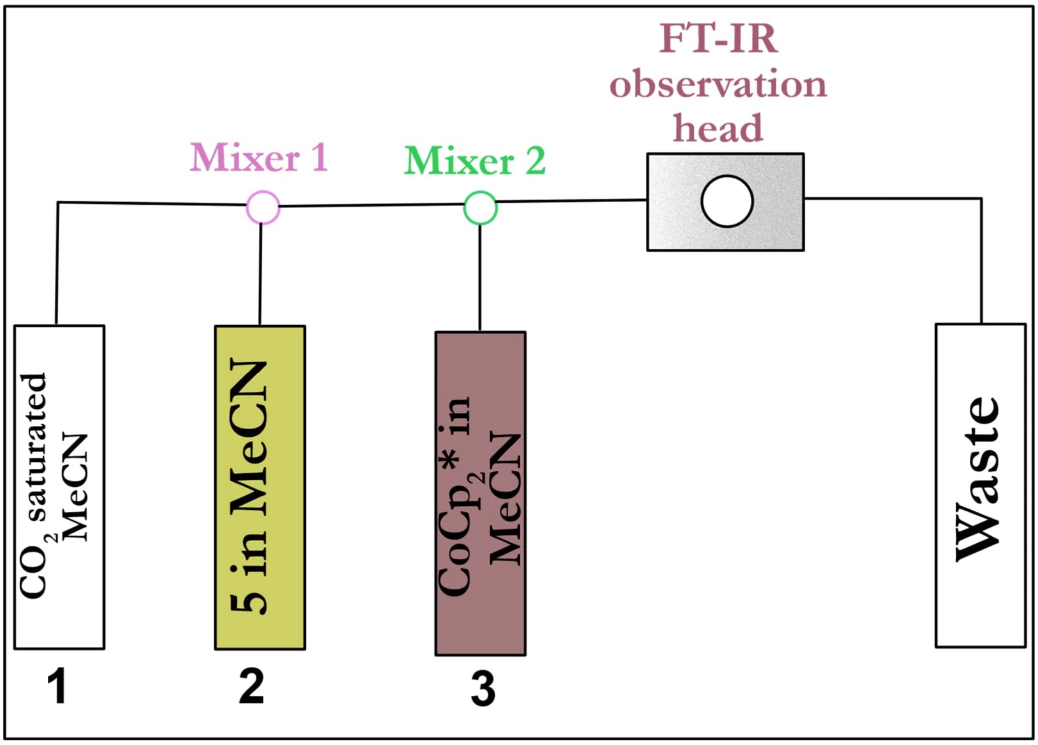

Figure S6: Schematic representation of the mixing sequence used during the stopped-flow TRIR experiments.

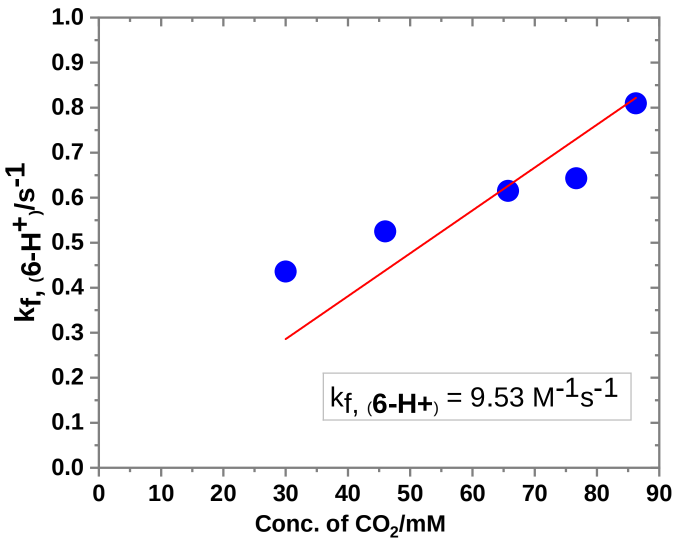

Figure S7: The plot of the formation rate constant of Mn-formate versus [CO_2] in anhydrous acetonitrile. The second-order rate constant (in $M^{-1}s^{-1}$) was calculated from the corresponding slopes of the linear fitting (using the equation y = mx). The goodness of the fits was measured by mean square error analysis, which is 0.1.

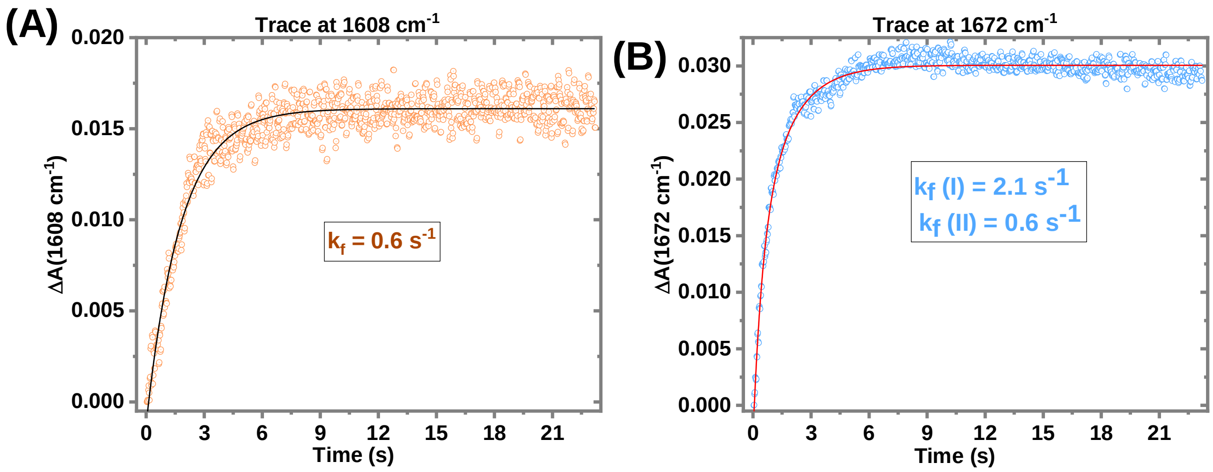

Figure S8: Kinetic traces at (**A**) 1608 cm^{-1}, and **(B**) 1672 cm^{-1} observed during the reaction of 0.5 mM **5** and 2.6 mM $CoCp_2$* in CO_2-saturated anhydrous acetonitrile. The rate constant values are mean values obtained from at least three individual experiments.

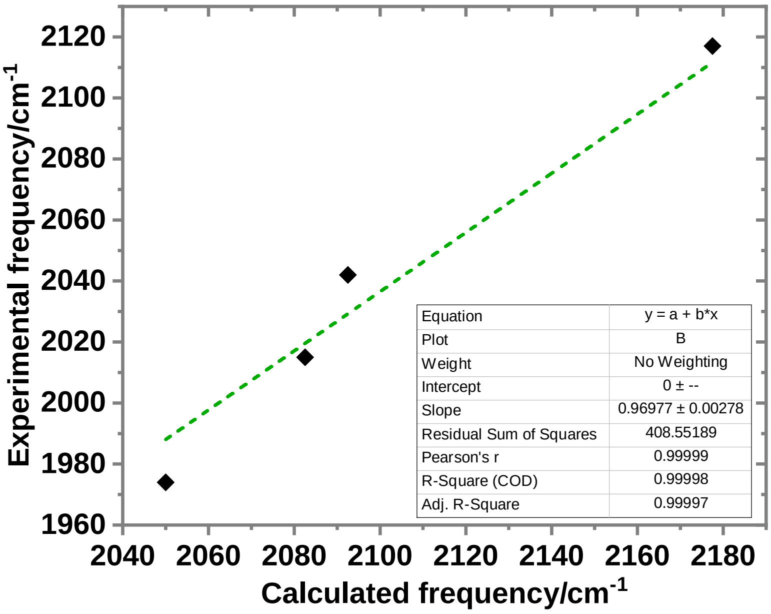

Figure S9: Correlation between the experimental and DFT calculated $\bar{\upsilon}_{CO}$ bands of **5** in acetonitrile. The slope of the linear fitting was used as a scaling factor for all the calculated frequencies shown in this work.

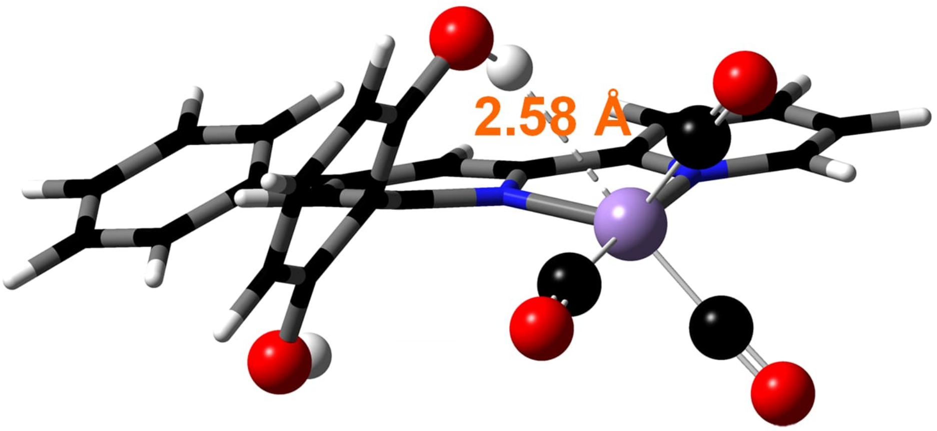

Figure S10: DFT optimized (b3lyp/def2-tzvp with GD3BJ and CPCM/MeCN) geometry of $[Mn(dhbpy)(CO)_3]^-$ species (**2**$^-$). The H-bonding distance from one of the pendant O-Hs to the reduced Mn (Mn^{-1}) center is shown here.

Table ST1: Experimental and Calculated (b3lyp/Def2-tzvp with GD3BJ dispersion correction) vibrational frequencies of the species discussed in this paper. [a, b] dhbpy is the ligand backbone (*Figure S8B*).

Species	CO vibrational frequencies ($\bar{\upsilon}_{CO}$/cm^{-1}) in acetonitrile	
	Experimental	Calculated
$Mn(dhbpy)(CO)_4SbF_6$ **(5)**	2117, 2042, 2015, 1974	2112, 2029, 2019, 1988
$Mn(dhbpy\text{-}H^+)(CO)_4SbF_6$ **(5-H$^+$)**	2111[a]	2090, 2017, 1992, 1961
$[Mn(dhbpy)(CO)_3]^-$ **(2$^-$)**	1916, 1822	1894, 1823, 1803
$[Mn(dhbpy\text{-}H^+)(CO)_3]^{2-}$ **(2$^-$-H$^+$)**	1910, 1818[b]	1874, 1801, 1774
$[HMn(dhbpy\text{-}H^+)(CO)_3]^-$ **(2H-H$^+$)**	1988[a]	1978, 1886, 1872
$[HMn(dhbpy)(CO)_3]$ **(2H)**	-	1993, 1903, 1893
$[Mn(dhbpy)(CO)_3COOH]$ **(4)**	2008[a]	2012, 1930, 1908
$[Mn(dhbpy)(CO)_3OCHO]$ **(6)**	-	2030, 1942, 1927
$[Mn(dhbpy\text{-}H^+)(CO)_3OCHO]$ **(6-H$^+$)**	2024, 1943, 1916	2024, 1940, 1915

[a] higher υ_{CO} band of the intermediates. The lower υ_{CO} bands overlap with those of other intermediates. [b] υ_{CO} vibration obtained from Ref. 24.

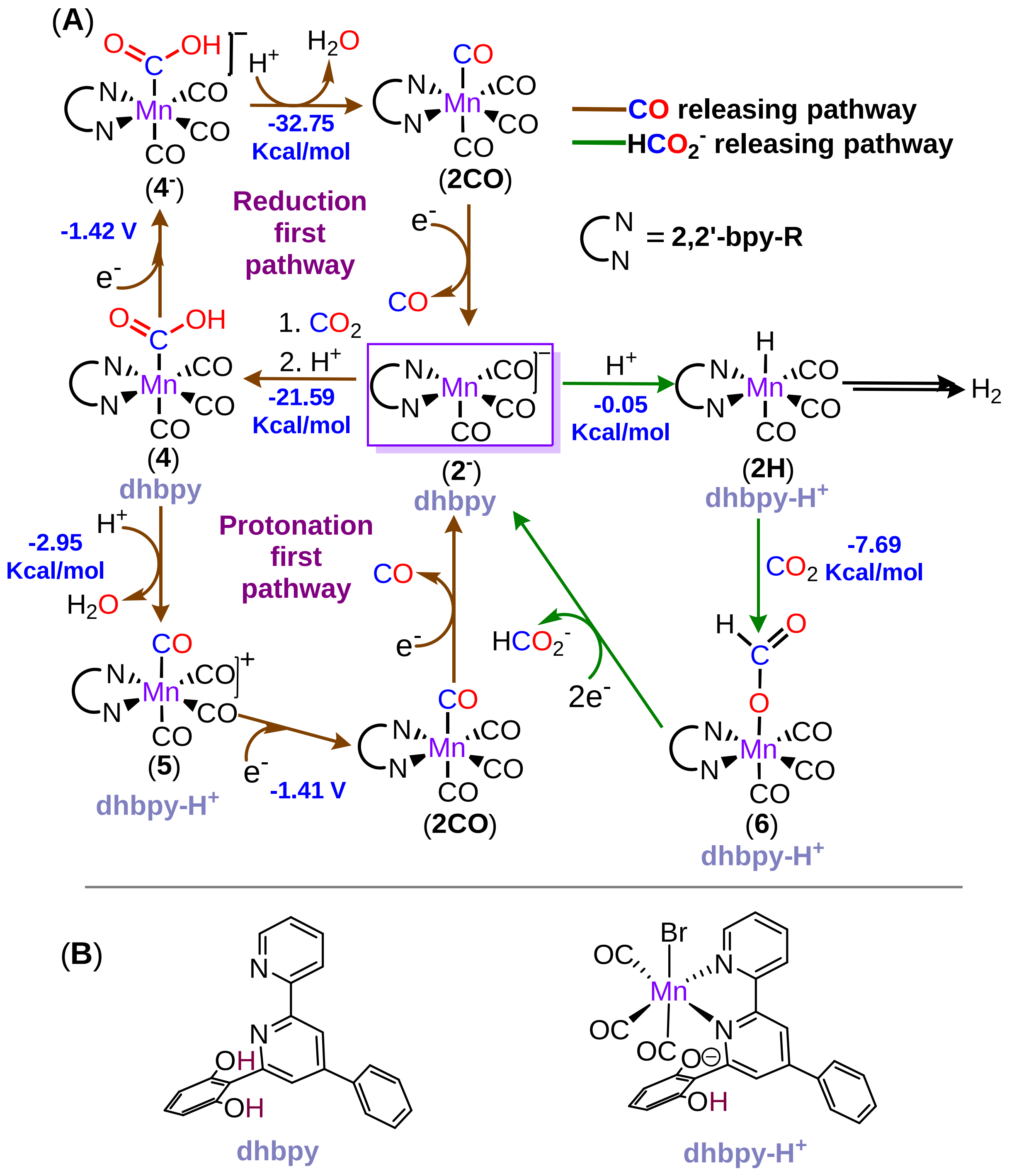

Figure S11: (A) Proposed catalytic cycle of CO_2 to CO and HCOOH production using the Mn-catalyst used in this study. Both the protonation-first and reduction-first pathways towards the CO formation are shown here. DFT calculated change in the standard reaction free energies and the calculated standard reduction potentials (presented with reference to SHE) are shown in blue. (**B**) Chemical structures of the ligand (protonated and deprotonated form).

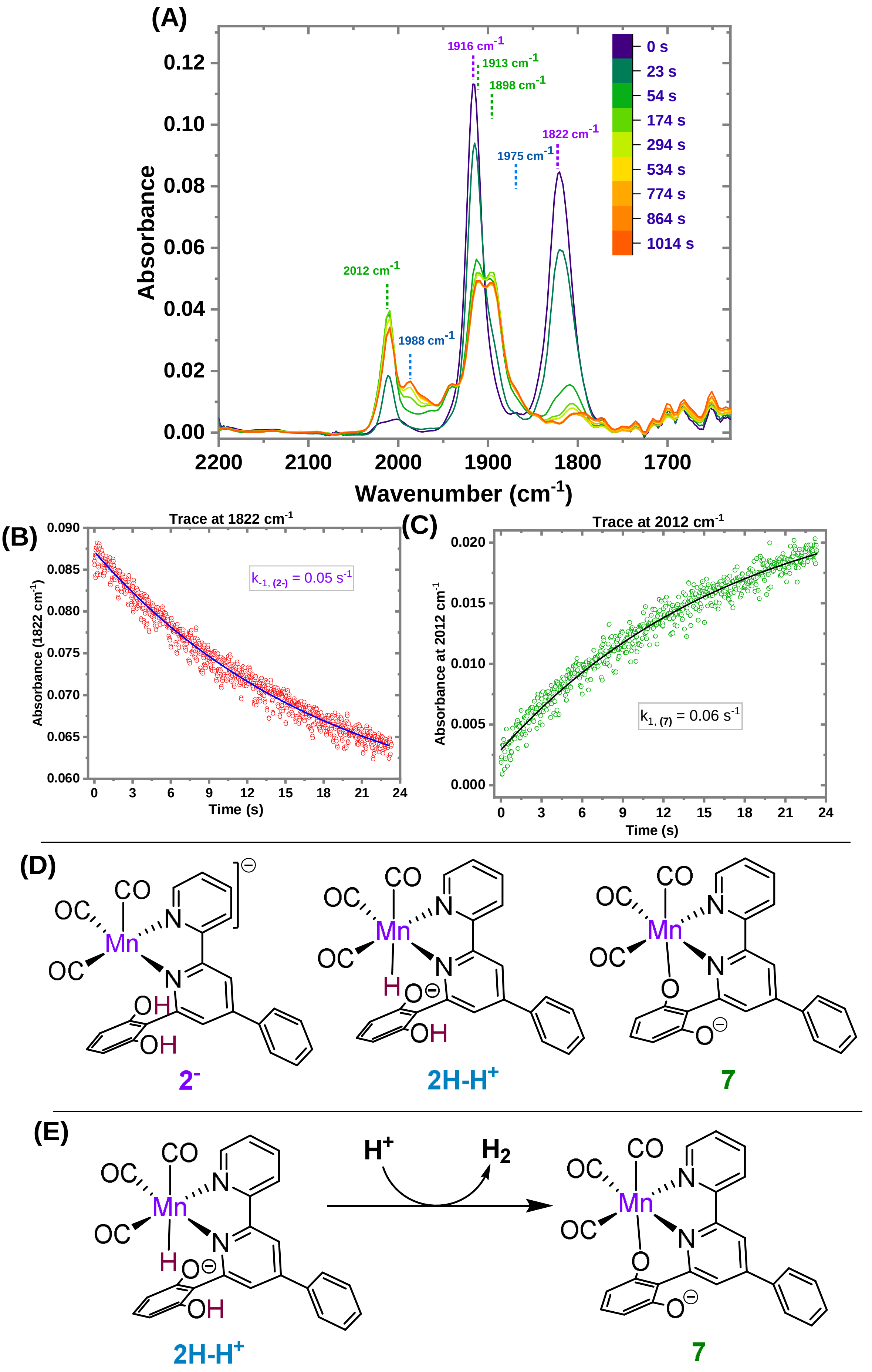
(A)
0 s
23 s
54 s
174 s
294 s
534 s
774 s
864 s
1014 s
1916 cm-1
1913 cm-1
1898 cm-1
1822 cm-1
1975 cm-1
2012 cm-1
1988 cm-1
Absorbance
Wavenumber (cm-1)
(B)
Trace at 1822 cm-1
k-1, (2-) = 0.05 s-1
Absorbance (1822 cm-1)
Time (s)
(C)
Trace at 2012 cm-1
k1, (7) = 0.06 s-1
Absorbance at 2012 cm-1
Time (s)
(D)
2-
2H-H+
7
(E)
H+
H2
2H-H+
7

Figure S12: (A) TRIR spectra of 1 mM of complex **5**, showing spectral changes upon adding 3 mM $CoCp_2$* in anhydrous acetonitrile under argon. Kinetic traces of **(B) 2**$^-$ (red) and **(C) 7** (green). **(D)** Chemical structures of the intermediates, **2**$^-$, **2H-H**$^+$, and **7**. (E) Plausible reaction happening under an argon atmosphere, producing H_2. The rate constant values are mean values obtained from at least three individual experiments.

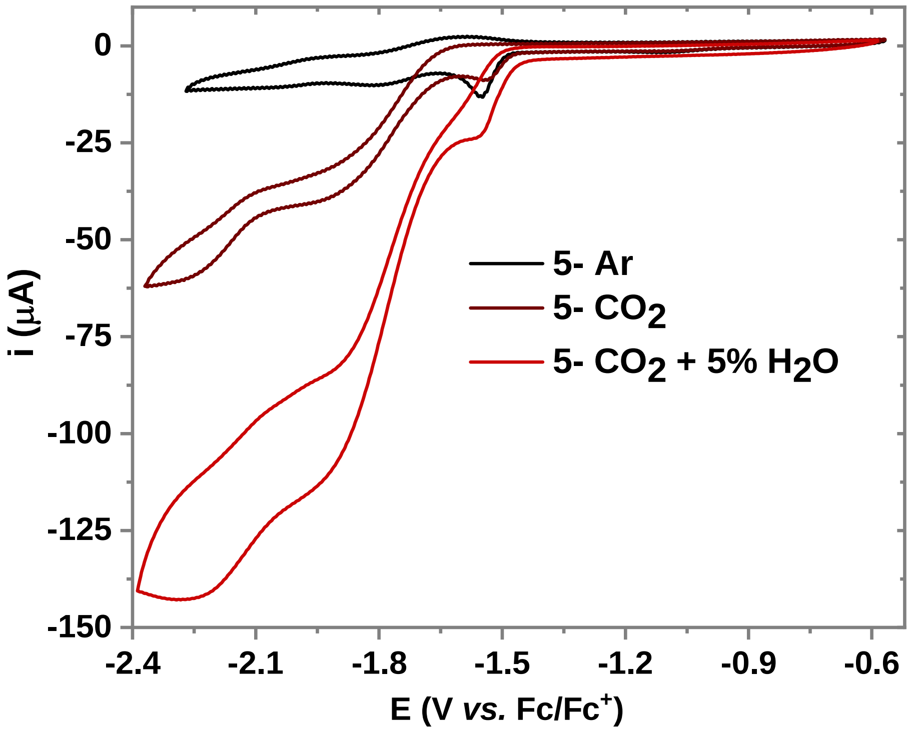

Figure S13: Cyclic voltammograms of **5** (1 mM) in acetonitrile under argon (black), and CO_2 atmosphere without and with 5% H_2O. Scan rate: 100 mV/s, working electrode: glassy carbon, counter electrode: Pt. Here, tetra-butyl ammonium hexafluoro phosphate was used as a supporting electrolyte.

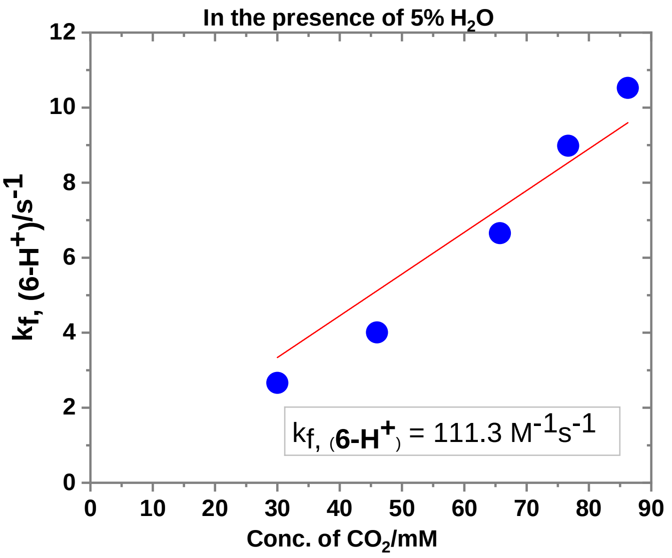

Figure S14: The plot of the formation rate constant of Mn-formate versus [CO_2] in the presence of 5% H_2O as the external proton source. The second-order rate constant (in $M^{-1}s^{-1}$) was calculated from the corresponding slopes of the linear fitting (using the equation y = mx). The goodness of the fits was measured by mean square error analysis, which is 0.9.

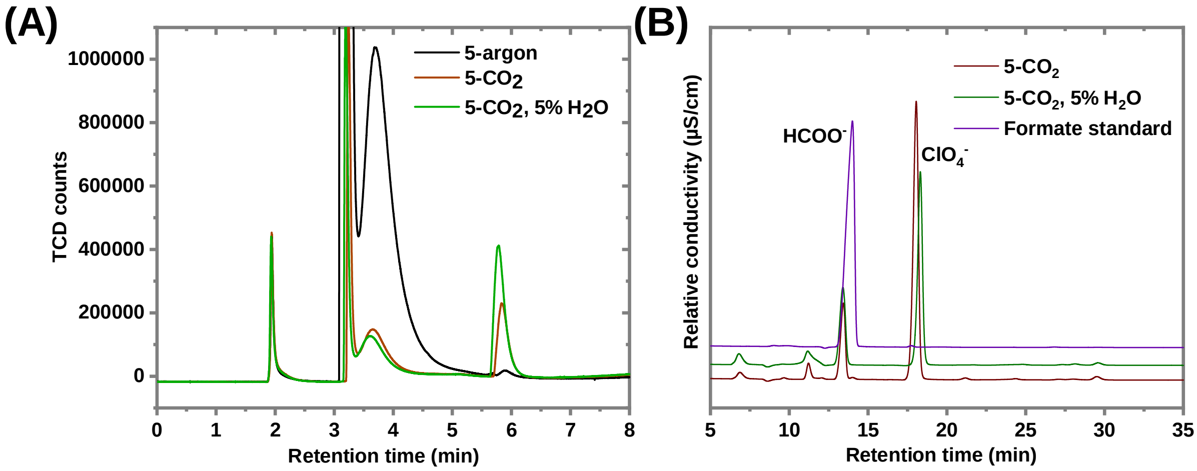

Figure S15: Product analysis after CPE at -1.9 V (*vs.* Fc/Fc^+) for 2 hrs. in the presence of 1 mM **5** by (**A**) GC-TCD: under argon (black), under CO_2 (brown), and CO_2 in the presence of 5% H_2O (green) and (**B**) ion-chromatography: under CO_2 (brown), under CO_2 in the presence of 5% H_2O (green) and the chromatogram of pure $HCOO^-$ is shown in purple.

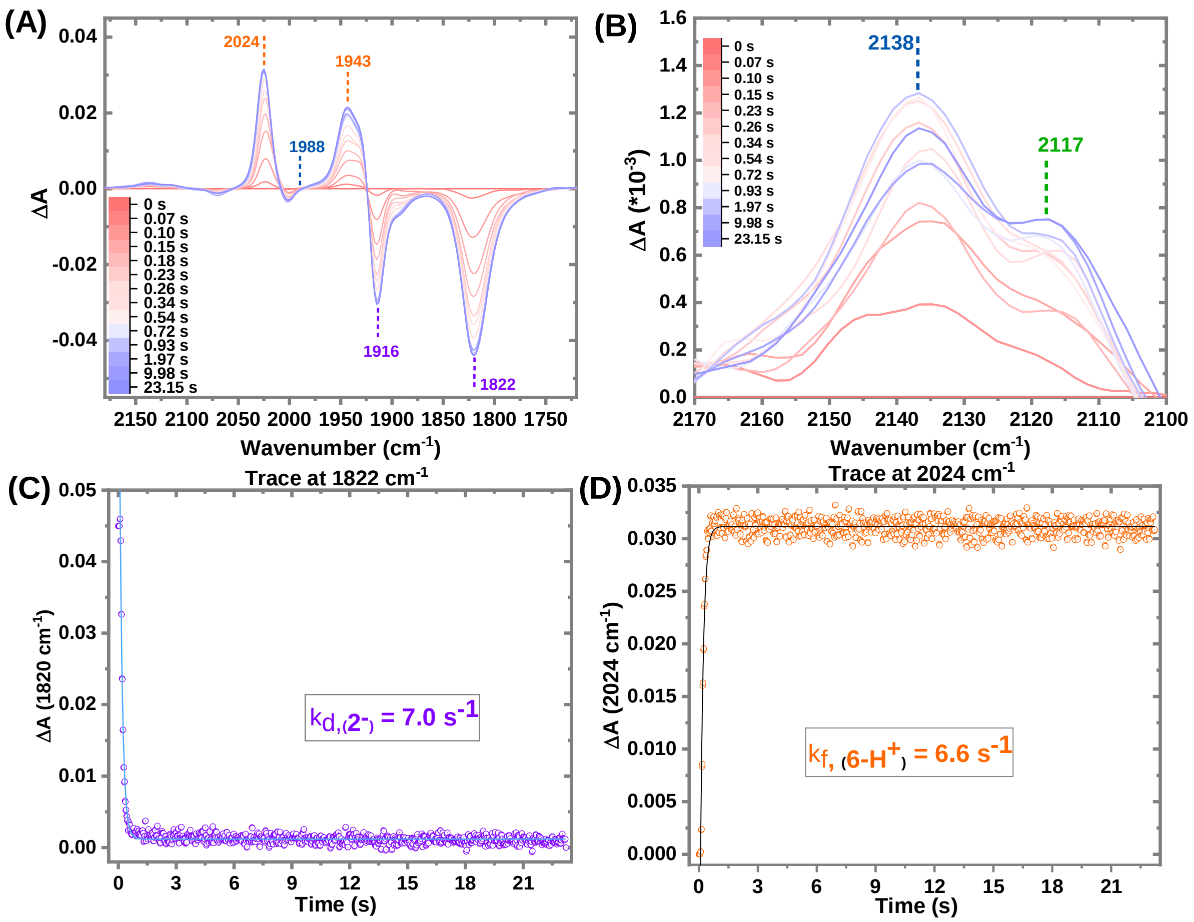

Figure S16: (A) TRIR difference spectra of 0.5 mM of complex **5** in acetonitrile showing the spectral changes upon adding 2.6 mM $CoCp_2*$ in CO_2-saturated acetonitrile containing 5% D_2O as the external proton source. **(B)** Zoomed-in section of (A). Kinetic traces of **(C)** **2**$^-$ (purple), and **(D)** **6-H**$^+$ (orange) species. In these figures, the decay of the species present at the initial time (within the time resolution of the instrument) and the formation of new species are represented as negative bands and positive bands, respectively. The rate constant values are mean values obtained from at least three individual experiments.

- *Optimized Coordinates:*

*1. [Mn(dhbpy)(CO)$_4$]$^+$ (**5**) :*

C	-2.22467000	-1.43938700	0.83181900
C	-3.53643600	-1.00289900	0.80385200
C	-3.78250800	0.35821300	0.71173600
C	-2.70851300	1.22956300	0.65594400
C	-1.41175800	0.72594200	0.69246200
H	-2.00367900	-2.49239200	0.90044800
H	-4.33901000	-1.72372600	0.85341500
H	-4.79363300	0.73901600	0.68590800

H	-2.88397200	2.29075400	0.58934000
C	-0.21235700	1.57552900	0.65725000
C	-0.30074700	2.95256000	0.52040300
C	0.84439100	3.74141600	0.50658100
H	-1.26808700	3.41932500	0.44165400
C	2.10023400	1.68899900	0.80102500
C	2.05676200	3.06923100	0.66354500
H	2.99152400	3.60909200	0.67444400
N	-1.18348100	-0.60017000	0.77623500
N	0.97594800	0.93647500	0.77598300
C	0.41850900	-2.96279800	0.73248300
C	0.76677600	-1.19515300	2.65940800
C	2.58081100	-1.69031900	0.73965500
O	0.70825700	-1.23570300	3.78894900
O	0.19459700	-4.07995800	0.70763600
O	3.60863900	-2.17747100	0.69740700
C	3.42871900	1.05843200	0.99966200
C	4.31973800	0.91031900	-0.07327800
C	3.86643000	0.72224800	2.28906400
C	5.58891700	0.38234100	0.12754900
C	5.13248800	0.19120000	2.49487700
C	5.97850800	0.02020800	1.40852500
H	6.24852900	0.25995600	-0.71977700
H	5.43848800	-0.07003300	3.49789800
H	6.96556600	-0.39413500	1.56510700
O	3.07172300	0.88568700	3.38400000
O	3.98058200	1.24584200	-1.34808200
H	3.11166500	1.66692800	-1.38036200
H	2.30313000	1.43379700	3.18065000
C	0.77868700	5.20338800	0.35108200
C	1.73366300	6.02835100	0.95542900
C	-0.24050900	5.79258300	-0.40547500
C	1.66630100	7.40643800	0.81138400
H	2.51695600	5.59563000	1.56278100
C	-0.30006400	7.17018800	-0.55597900
H	-0.97435200	5.17208700	-0.90178500
C	0.65110500	7.98142500	0.05392300
H	2.40462300	8.03222300	1.29471700
H	-1.08706300	7.61028900	-1.15371900
H	0.60159700	9.05613800	-0.06061100
Mn	0.80578000	-1.17698400	0.77081900
C	0.77853700	-1.10936000	-1.11629700
O	0.76001400	-1.07301800	-2.24750900

2. Mn(dhbpy-H$^+$)(CO)$_4$SbF$_6$ (**5-H$^+$**):

C	-2.20990700	-1.44224100	0.95874200
C	-3.52341700	-1.01390100	1.00864500

C	-3.78273600	0.34667900	0.92750600
C	-2.71969300	1.22448800	0.80824100
C	-1.41937800	0.72857100	0.76953100
H	-1.97531700	-2.49350200	1.01741700
H	-4.31800700	-1.73848500	1.10848600
H	-4.79640900	0.72061800	0.96048600
H	-2.90157600	2.28564100	0.75237600
C	-0.22525100	1.57994100	0.67332600
C	-0.31060400	2.95333500	0.52130400
C	0.84954300	3.72289700	0.45178000
H	-1.27479000	3.43177900	0.47216500
C	2.09916800	1.65990400	0.75642400
C	2.05548900	3.04352300	0.58597200
H	2.99247200	3.58021900	0.57363600
N	-1.17916100	-0.59676500	0.83836700
N	0.96093600	0.93352300	0.75418400
C	0.45941000	-2.93148700	0.58753800
C	0.84753700	-1.26986300	2.59451900
C	2.60086600	-1.60168600	0.48574200
O	0.68131300	-1.49699500	3.69165100
O	0.24975900	-4.05368800	0.52411200
O	3.61512400	-2.08441300	0.28807200
C	3.39912100	1.01834200	0.99752500
C	4.41952700	1.07495700	0.04144200
C	3.64566500	0.48929200	2.31896700
C	5.68964400	0.58815700	0.31033700
C	4.97259600	0.01970000	2.56052500
C	5.94385800	0.06943100	1.58378400
H	6.44817000	0.60873000	-0.45949200
H	5.18929800	-0.38817200	3.54046900
H	6.93608800	-0.30936600	1.80245000
O	2.72748000	0.45729100	3.20286900
O	4.20352700	1.57972100	-1.21895300
H	3.27992800	1.84328200	-1.31822600
C	0.79571500	5.18574700	0.27442300
C	1.75808300	6.01292700	0.86300600
C	-0.21870200	5.77292900	-0.48931700
C	1.70483800	7.38942100	0.69464100
H	2.53793800	5.58143300	1.47584500
C	-0.26603900	7.14897900	-0.66331200
H	-0.96033500	5.14993200	-0.97119800
C	0.69410600	7.96212000	-0.07045400
H	2.45064800	8.01585900	1.16594500
H	-1.05092700	7.58609900	-1.26634500
H	0.65479200	9.03523000	-0.20334300
Mn	0.81250600	-1.15036900	0.67790000
C	0.67883700	-1.00605700	-1.17909300
O	0.60249100	-0.91919600	-2.30909200

3. $[Mn(dhbpy)(CO)_3]^-$ ($\mathbf{2}^-$):

C	-2.36943200	-1.03441900	-0.79541900
C	-3.57658200	-0.51018900	-1.17060400
C	-3.77251100	0.88388700	-1.07279500
C	-2.74501100	1.66264300	-0.61013400
C	-1.51787300	1.07275800	-0.23809600
H	-2.17893000	-2.09642600	-0.85650700
H	-4.35602800	-1.16373400	-1.53585100
H	-4.71275300	1.33070900	-1.36625500
H	-2.86545600	2.73389600	-0.54123300
C	-0.37377800	1.76826100	0.23744400
C	-0.37700400	3.13152000	0.53423200
C	0.74570100	3.77851000	1.01107100
H	-1.30381900	3.67324100	0.41883400
C	1.91003000	1.64103500	0.77286400
C	1.91797900	2.97664200	1.07443300
H	2.87287600	3.43967900	1.28044600
N	-1.34179800	-0.28247300	-0.32682400
N	0.74457400	0.94863700	0.45111100
C	0.69126200	-2.04797400	-1.10145000
C	-0.41647000	-2.19136800	1.31427500
C	1.94238500	-1.58582400	1.07540700
O	-0.98410400	-2.98782700	1.94544200
O	0.86699700	-2.77041700	-1.99709300
O	2.83695600	-2.10842800	1.59721900
C	3.21744600	0.94956600	0.68871400
C	3.66835000	0.40644300	-0.52620600
C	4.09269600	0.92202000	1.78342200
C	4.95164300	-0.11183000	-0.65041800
C	5.37563600	0.39734600	1.67131600
C	5.79939200	-0.10606600	0.44961100
H	5.26303400	-0.51958500	-1.60240200
H	6.01999500	0.38791900	2.53974700
H	6.79931100	-0.50989800	0.35667700
O	3.71740000	1.39557300	3.00859100
O	2.86605900	0.39756600	-1.62802300
H	1.93738500	0.30707100	-1.33099700
H	2.81785200	1.75102500	2.94415600
C	0.75801400	5.19589300	1.38492200
C	1.71277000	5.69611000	2.28552600
C	-0.17992100	6.10403800	0.86588300
C	1.72582000	7.03506600	2.65173300
H	2.43939500	5.02438700	2.72285900
C	-0.16877400	7.44034100	1.23621900
H	-0.91404900	5.76509100	0.14730600
C	0.78468700	7.91818200	2.13207000
H	2.47084400	7.38824100	3.35371800
H	-0.90232900	8.11621500	0.81470100

H	0.79436300	8.96174200	2.41803400
Mn	0.42525200	-0.99919700	0.30263300

4. *[Mn(dhbpy-H$^+$)(CO)$_3$]$^{2-}$* (**2$^-$-H$^+$**):

C	-2.40163600	-1.03337900	-0.76648200
C	-3.60660100	-0.50187600	-1.13922600
C	-3.78991200	0.89488700	-1.05525000
C	-2.74981800	1.66672300	-0.60548500
C	-1.52801300	1.06965500	-0.23295400
H	-2.22050200	-2.09747200	-0.82030200
H	-4.39379500	-1.15262200	-1.49335700
H	-4.72705900	1.34844400	-1.34835400
H	-2.85855100	2.73994900	-0.54694300
C	-0.37507100	1.76374400	0.23419400
C	-0.37911600	3.13063000	0.52198100
C	0.75010200	3.76517500	0.99956800
H	-1.30299400	3.67729800	0.40725300
C	1.91146700	1.62478200	0.77323900
C	1.91235100	2.96112300	1.08126400
H	2.85981100	3.40274700	1.34879100
N	-1.36288500	-0.28819200	-0.30991400
N	0.73983500	0.95135100	0.44360900
C	0.69485400	-2.00750800	-1.14140600
C	-0.42309100	-2.23148800	1.27627400
C	1.91999300	-1.60217400	1.07169900
O	-0.98172500	-3.06013900	1.88219400
O	0.87516000	-2.72545300	-2.04608600
O	2.80052500	-2.14549200	1.60032400
C	3.21923600	0.93855700	0.71686500
C	3.67154300	0.40579400	-0.49532300
C	4.09262600	0.96359500	1.86938900
C	4.96191700	-0.09164900	-0.65430500
C	5.41275100	0.45111300	1.66150200
C	5.82405900	-0.05110300	0.44176000
H	5.26375800	-0.49938500	-1.60965500
H	6.08998300	0.46419000	2.50856900
H	6.83548700	-0.42933600	0.33354100
O	3.71747800	1.41255800	3.00745900
O	2.85567700	0.38617800	-1.60541700
H	1.94112500	0.21815300	-1.30023200
C	0.76475600	5.18588000	1.37327500
C	1.69303600	5.67416900	2.30649800
C	-0.14282200	6.10367500	0.82074500
C	1.70870200	7.01308200	2.67342400
H	2.39538500	4.99148600	2.76584900
C	-0.12901400	7.44068100	1.19035400
H	-0.85464800	5.77085900	0.07711000
C	0.79741500	7.90697800	2.11983400
H	2.43228600	7.35823300	3.40143000

H	-0.83853600	8.12539300	0.74263200
H	0.80970800	8.95069600	2.40550400
Mn	0.40639100	-1.01593700	0.29071400

5. *[HMn(dhbpy)(CO)$_3$] (**2H**):*

C	-2.23283100	-1.41761800	0.40840000
C	-3.50809600	-1.00267200	0.07028300
C	-3.74932300	0.35555000	-0.08304600
C	-2.70358700	1.24152700	0.10381200
C	-1.44289400	0.75475800	0.44357100
H	-2.01510700	-2.46648400	0.53634300
H	-4.28905600	-1.73589800	-0.06927800
H	-4.73227500	0.71894900	-0.34834500
H	-2.86618100	2.29993900	-0.02429800
C	-0.26589600	1.61364900	0.62124200
C	-0.35327600	2.99723700	0.59241700
C	0.79339200	3.77693900	0.70481700
H	-1.31735000	3.47151200	0.50810200
C	2.03918300	1.70025400	0.82388400
C	2.00218600	3.09093100	0.78994400
H	2.93841000	3.62924200	0.78550400
N	-1.21678800	-0.56562500	0.59292900
N	0.90932900	0.96375600	0.79965800
C	0.34190000	-2.84412700	1.23222500
C	0.25308700	-0.80617900	2.95554100
C	2.37558500	-1.53302800	1.68167800
O	-0.00033500	-0.67562000	4.06836300
O	0.14703900	-3.97893300	1.20784900
O	3.38608300	-1.94753000	2.04375400
C	3.37037200	1.05439900	0.80837600
C	3.77986800	0.28769900	-0.29847000
C	4.30616800	1.32610300	1.81725800
C	5.09547000	-0.14601300	-0.40759300
C	5.61555600	0.87087700	1.71969700
C	6.00340300	0.15140900	0.59912600
H	5.38151100	-0.72877300	-1.27206400
H	6.30892900	1.08707400	2.52049900
H	7.02474100	-0.19717300	0.51839900
O	3.98298000	2.03561200	2.93695500
O	2.91213600	-0.01430200	-1.30335800
H	2.08515700	-0.40621900	-0.91832100
H	3.04511600	2.26858800	2.93394100
C	0.73832900	5.24967500	0.70094900
C	1.65053200	5.99318000	1.45724900
C	-0.22370200	5.92974000	-0.05292100
C	1.59786700	7.37990400	1.46327600
H	2.38829800	5.48399700	2.06293800

C	-0.26991400	7.31681000	-0.05202800
H	-0.92263200	5.37403000	-0.66349100
C	0.63884100	8.04631000	0.70750200
H	2.30318400	7.94055800	2.06240200
H	-1.01288900	7.82861600	-0.64936500
H	0.60024500	9.12763900	0.71002800
Mn	0.68999800	-1.08831400	1.20200700
H	1.02378800	-1.35774300	-0.36160700

6. *[HMn(dhbpy-H^+)$(CO)_3$]* (**2H-H^+**):

C	-2.23449900	-1.31618500	1.07111500
C	-3.52281500	-0.88433200	1.32363600
C	-3.76115700	0.48115700	1.42330500
C	-2.69985900	1.35420200	1.27057200
C	-1.42506800	0.85027800	1.01410900
H	-2.01070400	-2.36955800	0.99289400
H	-4.31636000	-1.60764800	1.44335700
H	-4.75421000	0.85768000	1.62600800
H	-2.85483300	2.41785500	1.36450900
C	-0.23745200	1.69564200	0.85655400
C	-0.32964900	3.05651500	0.63903100
C	0.83161300	3.81921300	0.48197900
H	-1.29723000	3.52964400	0.58811300
C	2.09029000	1.77810300	0.86233600
C	2.03940200	3.15193300	0.61202500
H	2.97731500	3.67466900	0.51848300
N	-1.20315100	-0.47506900	0.91309800
N	0.95416300	1.04314100	0.91339500
C	0.25029400	-2.57009500	-0.27363800
C	0.96360800	-1.84174700	2.12703600
C	2.41606600	-1.36480600	-0.07003700
O	1.08381700	-2.49609600	3.06916400
O	-0.06927800	-3.53151300	-0.82771800
O	3.43106200	-1.66856000	-0.52036700
C	3.39395300	1.16470500	1.13432600
C	4.48319200	1.39043600	0.20873900
C	3.61309100	0.48577900	2.34110900
C	5.75561500	0.87238700	0.60900000
C	4.86396400	-0.00819600	2.68681800
C	5.92454700	0.19882600	1.80040900
H	6.59001300	1.02056000	-0.06648600
H	4.99571600	-0.52190900	3.62897800
H	6.90630300	-0.18147600	2.06175300
O	2.61413000	0.33497400	3.27116300
O	4.31260700	2.00063400	-0.89753300
H	1.75926900	0.47284600	2.83951900
C	0.76961800	5.26979100	0.21518600
C	1.74859600	6.13280900	0.71930900

C	-0.26667800	5.81138000	-0.55297900
C	1.69170800	7.49607100	0.46429400
H	2.54773300	5.73801900	1.33212900
C	-0.31947900	7.17410900	-0.81310800
H	-1.02328300	5.16051700	-0.97066700
C	0.65852600	8.02220600	-0.30432200
H	2.45298400	8.14916000	0.87068000
H	-1.12286000	7.57309900	-1.41858100
H	0.61583900	9.08466700	-0.50509600
Mn	0.73375400	-1.04317300	0.49921800
H	0.49031400	-0.50016900	-0.99360200

7. *[Mn(dhbpy)(CO)$_3$COOH]* (**4**):

C	-2.22832700	-1.44417700	0.87952400
C	-3.53540500	-1.01367100	1.01581400
C	-3.78461900	0.34997600	1.08224000
C	-2.71673800	1.22717300	1.00958200
C	-1.42602400	0.72573200	0.86463100
H	-1.99967600	-2.49825100	0.83763300
H	-4.33339500	-1.73923900	1.07434600
H	-4.79195100	0.72487000	1.19697000
H	-2.88491100	2.29026700	1.08000900
C	-0.23084000	1.57454700	0.79331500
C	-0.31083900	2.94152200	0.59122900
C	0.84785000	3.71137300	0.50893100
H	-1.27553700	3.41225700	0.49329200
C	2.08142200	1.66197200	0.88431800
C	2.05277300	3.03679500	0.67923900
H	2.99390200	3.56393600	0.63947800
N	-1.19101500	-0.59980300	0.79956700
N	0.95388100	0.92687000	0.90991100
C	0.42871800	-2.86896400	0.22622700
C	0.82263800	-1.55208200	2.46032200
C	2.54028400	-1.65672800	0.47526300
O	0.83781500	-1.83437900	3.56961500
O	0.20855400	-3.96013200	-0.06314600
O	3.57941000	-2.11846000	0.32515200
C	3.40196500	1.03271400	1.12657900
C	4.33410700	0.90272300	0.08516700
C	3.79260500	0.70562900	2.43135400
C	5.64083100	0.51152700	0.35806200
C	5.09006500	0.28641100	2.70186300
C	6.00668500	0.21031500	1.66235000
H	6.34020200	0.41383700	-0.46060100
H	5.36494600	0.04176600	3.71838200
H	7.01991400	-0.10744000	1.87164200
O	2.93796800	0.80686800	3.48935100
O	3.98147400	1.14406500	-1.20091400

H	3.05320600	0.83610800	-1.37593300
H	2.05857600	1.07786500	3.19660500
C	0.79582300	5.16361800	0.26210000
C	1.75545100	6.01689800	0.81672000
C	-0.21223400	5.71375600	-0.53687400
C	1.70517400	7.38364600	0.58166600
H	2.53100500	5.61442500	1.45430000
C	-0.25634600	7.07971500	-0.77746200
H	-0.95040900	5.06912400	-0.99493300
C	0.70066600	7.91952100	-0.21751400
H	2.44883000	8.03139600	1.02674100
H	-1.03584500	7.48779400	-1.40720800
H	0.66411900	8.98485500	-0.40315900
Mn	0.80217900	-1.16605200	0.66016300
C	0.80425100	-0.59082100	-1.33588700
O	-0.22233900	-0.97002500	-2.16524500
O	1.62848300	0.12731800	-1.89217300
H	-0.84864500	-1.50487900	-1.66216900

8. *[Mn(dhbpy)(CO)$_3$OCHO]* (**6**):

C	-2.27070700	-1.41274600	0.52621800
C	-3.55957500	-0.98811500	0.25565300
C	-3.79262600	0.37026100	0.10454900
C	-2.72975800	1.24846600	0.22877300
C	-1.45770800	0.75313000	0.50144900
H	-2.05911900	-2.46284300	0.65331600
H	-4.35446600	-1.71392300	0.16696200
H	-4.78464300	0.74296700	-0.10880800
H	-2.89265900	2.30717300	0.10669900
C	-0.26780600	1.60941500	0.62670500
C	-0.35222900	2.99204100	0.57269000
C	0.79743100	3.77216500	0.65644000
H	-1.31512700	3.46845700	0.49017300
C	2.03897900	1.69818000	0.80522600
C	2.00615300	3.08719100	0.75236600
H	2.94431600	3.62159500	0.75222900
N	-1.23971800	-0.56880200	0.64434400
N	0.91035200	0.96449600	0.78793400
C	0.33354300	-2.88555000	1.17096500
C	0.43238600	-0.90588400	2.82961300
C	2.44529700	-1.61760400	1.39361000
O	0.25386900	-0.76161400	3.95179700
O	0.11337000	-4.00599100	1.27161300
O	3.45455200	-2.08226100	1.66540700
C	3.37204100	1.05097700	0.83361000
C	3.90808600	0.47884500	-0.33475800
C	4.17938200	1.15203400	1.97356500
C	5.22989000	0.04601600	-0.35221800
C	5.49271100	0.69790700	1.96009400

C	6.00897100	0.15824500	0.79124000
H	5.62295700	-0.38736000	-1.26134900
H	6.08806200	0.77581800	2.85907100
H	7.03309200	-0.19133900	0.77531000
O	3.71929300	1.68919400	3.14040900
O	3.17612300	0.38268400	-1.47470200
H	2.30490100	-0.07723500	-1.31816400
H	2.79587800	1.95932100	3.05196100
C	0.74259700	5.24410900	0.62290200
C	1.66569800	6.00297300	1.35020400
C	-0.22966300	5.90746700	-0.13282000
C	1.61344200	7.38935200	1.32624700
H	2.41123300	5.50710500	1.95726400
C	-0.27461400	7.29408600	-0.16267400
H	-0.93694800	5.33920600	-0.72171500
C	0.64471200	8.03920500	0.56841200
H	2.32683000	7.96277100	1.90322200
H	-1.02493000	7.79318200	-0.76146800
H	0.60681300	9.12034200	0.54731300
Mn	0.71384000	-1.11938800	1.05559800
H	-0.11125400	-2.65957400	-1.50504100
O	1.03546800	-1.08316600	-0.97583700
C	0.55269200	-1.85965200	-1.88205700
O	0.77364000	-1.76614600	-3.08277700

9. *[Mn(dhbpy-H$^+$)(CO)$_3$OCHO]$^-$* (***6-H$^+$***):

C	-2.13046500	-1.47057100	1.13272600
C	-3.42983700	-1.05262000	1.35175800
C	-3.68953700	0.31086600	1.40159000
C	-2.64125100	1.19939400	1.23960800
C	-1.35509000	0.70991700	1.02338700
H	-1.88824400	-2.52173600	1.08559700
H	-4.21418400	-1.78396200	1.48055200
H	-4.69226600	0.67675500	1.57363700
H	-2.81581800	2.26245800	1.29856600
C	-0.17467500	1.57185200	0.87121000
C	-0.28029600	2.91679800	0.58540900
C	0.87802700	3.68695500	0.42551100
H	-1.25155600	3.37246300	0.47719700
C	2.15343700	1.68927500	0.94540500
C	2.09181900	3.04831400	0.62195700
H	3.02444900	3.57925000	0.52425100
N	-1.11539900	-0.61439300	0.96541100
N	1.02366500	0.94827100	1.00738800
C	0.43045300	-2.76180100	-0.11270700
C	1.05918700	-1.85703900	2.16210700
C	2.57063900	-1.44967400	0.04366500
O	1.13981400	-2.41149600	3.16348400

O	0.18897400	-3.80680400	-0.52102900
O	3.60596400	-1.77310000	-0.31673200
C	3.44705200	1.09025100	1.27648200
C	4.56531400	1.28588400	0.37702600
C	3.62434700	0.43029400	2.50255300
C	5.81541200	0.74334100	0.81454200
C	4.85413800	-0.08737300	2.88253500
C	5.93902300	0.08063300	2.01634700
H	6.66843200	0.86377600	0.15738900
H	4.95539500	-0.58151700	3.83868500
H	6.90484000	-0.31911300	2.30604600
O	2.60579500	0.32020400	3.41599000
O	4.43508000	1.88346800	-0.73959400
H	1.77240300	0.57993500	3.00063300
C	0.80249700	5.12004600	0.08093100
C	1.74684200	6.02566900	0.57588400
C	-0.21203300	5.59993400	-0.75428700
C	1.67700000	7.37226600	0.24682400
H	2.52701400	5.67858000	1.23972600
C	-0.27661400	6.94562700	-1.08900300
H	-0.93992100	4.91349200	-1.16588000
C	0.66630200	7.83718100	-0.58822900
H	2.41024200	8.06027500	0.64694400
H	-1.06154700	7.29724700	-1.74576600
H	0.61377000	8.88660200	-0.84686100
Mn	0.83027900	-1.12452900	0.52490400
H	-1.14090700	-1.31515300	-1.80437300
O	0.51289600	-0.25182700	-1.32670600
C	-0.45505100	-0.50279000	-2.12030500
O	-0.68069400	0.07893400	-3.18312700

10. [Mn(dhbpy)(CO)$_3$]$^-$ (**2**$^-$) with one H_2O molecule:

C	-1.99320000	-0.32881900	1.41550200
C	-3.20991700	0.29748200	1.38597900
C	-3.25014100	1.69873800	1.23378400
C	-2.06894300	2.38257700	1.11375100
C	-0.84129500	1.68843100	1.14600800
H	-1.91998700	-1.40086300	1.52918200
H	-4.11584200	-0.28468600	1.47736000
H	-4.19532400	2.22354000	1.20407000
H	-2.07413300	3.45467900	0.98335600
C	0.44707200	2.27567800	1.01030500
C	0.65212300	3.65171100	0.92121100
C	1.91636200	4.19679200	0.79345500
H	-0.20879800	4.29937000	0.98831600
C	2.74637000	1.89800300	0.72646400
C	2.96679800	3.24884800	0.66236700
H	3.96044200	3.58428900	0.40060400

N	-0.81251000	0.33042000	1.30799900
N	1.49968500	1.34983600	1.01369200
C	0.65096000	-1.82346400	0.26569100
C	0.50893600	-1.38909900	2.88484800
C	2.65308000	-1.17095200	1.73160500
O	0.15953200	-1.97818500	3.82496400
O	0.42217500	-2.71101700	-0.45226500
O	3.63997300	-1.70648100	2.02119000
C	3.86797100	1.01066200	0.34088700
C	3.77590000	0.19462500	-0.79673500
C	5.09529300	1.05245300	1.01628200
C	4.86305500	-0.52929300	-1.26121500
C	6.19176600	0.32652700	0.56307500
C	6.07166500	-0.45143400	-0.57877400
H	4.74787600	-1.14630700	-2.14154700
H	7.12120300	0.37897300	1.11347900
H	6.92545400	-1.01296500	-0.93483500
O	5.26319700	1.79364300	2.15028000
O	2.59487900	0.11956200	-1.49589300
H	1.86496400	0.02816200	-0.83870700
H	4.43721600	2.25969000	2.34906900
C	2.16848100	5.64087600	0.74756200
C	3.41586600	6.16507900	1.12200000
C	1.17945300	6.54806100	0.33438600
C	3.66065400	7.53103100	1.08905100
H	4.19558600	5.49842000	1.46546900
C	1.42408800	7.91285300	0.30608300
H	0.21518200	6.18008700	0.01014700
C	2.66707100	8.41583100	0.68236500
H	4.63054900	7.90635300	1.39044000
H	0.64377000	8.58773600	-0.02253300
H	2.85790800	9.48062500	0.65674100
Mn	1.00393800	-0.52028900	1.41399800
O	1.71303100	2.72531100	-2.45638000
H	2.04362700	1.84556300	-2.20593800
H	1.70742300	3.21992800	-1.62704500

- References:

(1) Franco, F.; Cometto, C.; Ferrero Vallana, F.; Sordello, F.; Priola, E.; Minero, C.; Nervi, C.; Gobetto, R. A local proton source in a [Mn(bpy-R)(CO)3Br]-type redox catalyst enables CO2 reduction even in the absence of Brønsted acids. *Chemical Communications* 2014, *50* (93), 14670-14673, 10.1039/C4CC05563B. DOI: 10.1039/C4CC05563B.

(2) Kuo, H.-Y.; Tignor, S. E.; Lee, T. S.; Ni, D.; Park, J. E.; Scholes, G. D.; Bocarsly, A. B. Reduction-induced CO dissociation by a [Mn(bpy)(CO)4][SbF6] complex and its relevance in electrocatalytic CO2 reduction. *Dalton Transactions* 2020, *49* (3), 891-900. DOI: 10.1039/C9DT04150H.

(3) Smieja, J. M.; Sampson, M. D.; Grice, K. A.; Benson, E. E.; Froehlich, J. D.; Kubiak, C. P. Manganese as a Substitute for Rhenium in CO2 Reduction Catalysts: The Importance of Acids. *Inorganic Chemistry* 2013, *52* (5), 2484-2491. DOI: 10.1021/ic302391u.

(4) Costentin, C.; Drouet, S.; Robert, M.; Savéant, J.-M. A Local Proton Source Enhances CO2 Electroreduction to CO by a Molecular Fe Catalyst. *Science* 2012, *338* (6103), 90-94. DOI: 10.1126/science.1224581 (acccessed 2025/02/26).
(5) Sampson, M. D.; Nguyen, A. D.; Grice, K. A.; Moore, C. E.; Rheingold, A. L.; Kubiak, C. P. Manganese Catalysts with Bulky Bipyridine Ligands for the Electrocatalytic Reduction of Carbon Dioxide: Eliminating Dimerization and Altering Catalysis. *Journal of the American Chemical Society* 2014, *136* (14), 5460-5471. DOI: 10.1021/ja501252f.
(6) Frisch, M. J. e. a. Gaussian 09, Rev. D.01. *Gaussian, Inc., Wallingford CT* 2016.
(7) Miertuš, S.; Scrocco, E.; Tomasi, J. Electrostatic interaction of a solute with a continuum. A direct utilizaion of AB initio molecular potentials for the prevision of solvent effects. *Chemical Physics* 1981, *55* (1), 117-129. DOI: https://doi.org/10.1016/0301-0104(81)85090-2.
(8) Cossi, M.; Scalmani, G.; Rega, N.; Barone, V. New developments in the polarizable continuum model for quantum mechanical and classical calculations on molecules in solution. *The Journal of Chemical Physics* 2002, *117* (1), 43-54. DOI: 10.1063/1.1480445 (acccessed 2/26/2024).
(9) Becke, A. D. Density-functional thermochemistry. III. The role of exact exchange. *Journal of Chemical Physics* 1993, *98*, 5648-5652. DOI: 10.1063/1.464913.
(10) Lee, C.; Yang, W.; Parr, R. G. Development of the Colle-Salvetti correlation-energy formula into a functional of the electron density. *Physical Review B* 1988, *37* (2), 785-789. DOI: 10.1103/PhysRevB.37.785.
(11) Weigend, F.; Ahlrichs, R. Balanced basis sets of split valence, triple zeta valence and quadruple zeta valence quality for H to Rn: Design and assessment of accuracy. *Physical Chemistry Chemical Physics* 2005, *7* (18), 3297-3305, 10.1039/B508541A. DOI: 10.1039/B508541A.
(12) Weigend, F. Accurate Coulomb-fitting basis sets for H to Rn. *Physical Chemistry Chemical Physics* 2006, *8* (9), 1057-1065, 10.1039/B515623H. DOI: 10.1039/B515623H.
(13) Franco, F.; Cometto, C.; Nencini, L.; Barolo, C.; Sordello, F.; Minero, C.; Fiedler, J.; Robert, M.; Gobetto, R.; Nervi, C. Local Proton Source in Electrocatalytic CO2 Reduction with [Mn(bpy–R)(CO)3Br] Complexes. *Chemistry – A European Journal* 2017, *23* (20), 4782-4793. DOI: 10.1002/chem.201605546.
(14) Grimme, S.; Ehrlich, S.; Goerigk, L. Effect of the damping function in dispersion corrected density functional theory. *Journal of Computational Chemistry* 2011, *32* (7), 1456-1465. DOI: https://doi.org/10.1002/jcc.21759 (acccessed 2024/02/26).
(15) Rotundo, L.; Garino, C.; Priola, E.; Sassone, D.; Rao, H.; Ma, B.; Robert, M.; Fiedler, J.; Gobetto, R.; Nervi, C. Electrochemical and Photochemical Reduction of CO2 Catalyzed by Re(I) Complexes Carrying Local Proton Sources. *Organometallics* 2019, *38* (6), 1351-1360. DOI: 10.1021/acs.organomet.8b00588.
(16) Mondal, B.; Chattopadhyay, S.; Dey, S.; Mahammed, A.; Mittra, K.; Rana, A.; Gross, Z.; Dey, A. Elucidation of Factors That Govern the 2e–/2H+ vs 4e–/4H+ Selectivity of Water Oxidation by a Cobalt Corrole. *Journal of the American Chemical Society* 2020, *142* (50), 21040-21049. DOI: 10.1021/jacs.0c08654.
